# Barcode System for Genetic Identification of Soybean [*Glycine max* (L.) Merrill] Cultivars Using InDel Markers Specific to Dense Variation Blocks

**DOI:** 10.3389/fpls.2017.00520

**Published:** 2017-04-10

**Authors:** Hwang-Bae Sohn, Su-Jeong Kim, Tae-Young Hwang, Hyang-Mi Park, Yu-Young Lee, Kesavan Markkandan, Dongwoo Lee, Sunghoon Lee, Su-Young Hong, Yun-Ho Song, Bon-Cheol Koo, Yul-Ho Kim

**Affiliations:** ^1^Highland Agriculture Research Institute, National Institute of Crop Science, Rural Development Administration (RDA)Gangwon-do, South Korea; ^2^Grassland and Forages Division, National Institute of Animal Science, Rural Development Administration (RDA)Chungcheongnam-Do, South Korea; ^3^Headquarters, National Institute of Crop Science, Rural Development Administration (RDA)Jeolabuk-Do, South Korea; ^4^Department of Central Area, National Institute of Crop Science, Rural Development Administration (RDA)Gyeonggi-Do, South Korea; ^5^TheragenEtex Bio Institute, TheragenEtex Inc.Gyeonggi-Do, South Korea; ^6^EONE-DIAGNOMICS Genome CenterIncheon, South Korea; ^7^Gangwondo Agricultural Research and Extension ServicesGangwon-Do, South Korea

**Keywords:** barcode, genetic identification, InDel marker, soybean (*Glycine max)*, variation block

## Abstract

For genetic identification of soybean [*Glycine max* (L.) Merrill] cultivars, insertions/deletions (InDel) markers have been preferred currently because they are easy to use, co-dominant and relatively abundant. Despite their biological importance, the investigation of InDels with proven quality and reproducibility has been limited. In this study, we described soybean barcode system approach based on InDel makers, each of which is specific to a dense variation block (dVB) with non-random recombination due to many variations. Firstly, 2,274 VBs were mined by analyzing whole genome data in six soybean cultivars (Backun, Sinpaldal 2, Shingi, Daepoong, Hwangkeum, and Williams 82) for transferability to dVB-specific InDel markers. Secondly, 73,327 putative InDels in the dVB regions were identified for the development of soybean barcode system. Among them, 202 dVB-specific InDels from all soybean cultivars were selected by gel electrophoresis, which were converted as 2D barcode types according to comparing amplicon polymorphisms in the five cultivars to the reference cultivar. Finally, the polymorphism of the markers were assessed in 147 soybean cultivars, and the soybean barcode system that allows a clear distinction among soybean cultivars is also detailed. In addition, the changing of the dVBs in a chromosomal level can be quickly identified due to investigation of the reshuffling pattern of the soybean cultivars with 27 maker sets. Especially, a backcross-inbred offspring, “Singang” and a recurrent parent, “Sowon” were identified by using the 27 InDel markers. These results indicate that the soybean barcode system enables not only the minimal use of molecular markers but also comparing the data from different sources due to no need of exploiting allele binning in new varieties.

## Introduction

The task of plant variety and cultivar identification is vital from breeding to cultivar registration, seed production, trade, and inspection. Crop identification can be usually addressed via two strategies, morphological descriptors and molecular markers (Inger and Rodomiro, 2000; Agarwal et al., 2008; Korir et al., 2013). Although morphological descriptors are traditionally used crop identification approach for testing distinctness, uniformity, and stability (DUS), but, their utility is less suitable when results are required rapidly in large collections or breeding lines with narrow genetic diversity. To date, researchers have been focused on incorporating molecular markers for this purpose, and rely on the availability of an adequate genetic marker collection in order to provide a high discrimination power. Recently, with the advent of new next generation sequencing (NGS) platforms, large volumes of sequencing data are being generated that could be screened with the aid of bioinformatics tools for exploiting molecular markers, including simple sequence repeats (SSRs), single nucleotide polymorphisms (SNPs), and insertion/deletions (InDels) for genetic study in crop plants (Ganal et al., 2009; Deschamps and Campbell, 2010; Hyten et al., 2010b; Kim et al., 2010; Song et al., 2010; Liu et al., 2012; Li et al., 2014; Moghaddam et al., 2014).

Soybean [*Glycine max* (L.) Merrill] genotyping is currently based on SSRs and SNPs which have been very useful not only for genetic identification but also for high-density genetic mapping (Hwang et al., 2009; Hyten et al., 2010a; Song et al., 2010, 2013; Lee et al., 2015). The universal use of SSRs leads to the establishment of BARCSOYSSR database (33,065 SSRs) and development of simple typing methodologies (Song et al., 2010). However, the SSR-based genotyping in common laboratories has some limitations as follows; a large amount of time and labor would be required because of using polyacrylamide electrophoresis; technical artifacts, such as different allele sizes and consequently different bins depending on analytical systems, would add ambiguity to inter-laboratory analysis; and the relatively high mutation rate of STR loci (~10^−3^) would confound the genetic identification of soybean varieties with non-redundant genotypes. Further, SNPs overcome some of the limitations of SSRs, such as genotyping errors resulting from stutter bands, technical artifacts, and a high mutation rate (Pompanon et al., 2005). In addition, SNP-based genotyping is usually complex, expensive, platform-dependent, and hard to be conducted in common laboratories (Lee et al., 2015).

For genetic study, InDel markers are gaining more attention among the molecular breeding scientists because they are easy to use, PCR-based, co-dominant (fully informative), and relatively abundant (Hou et al., 2010; Mullaney et al., 2010; Pacurar et al., 2012; Montgomery et al., 2013; Yamaki et al., 2013; Moghaddam et al., 2014; Wu et al., 2014). Notably, these markers are also readily accessible; either as designed and tested PCR markers deposited at SoyBase (http://SoyBase.org) or as polymorphisms identified in direct sequence comparisons with the development of resource-efficient NGS technology (Kim et al., 2010; Lam et al., 2010; Li and Durbin, 2010; Song et al., 2015; Zhou et al., 2015). Diverse soybean cultivars/varieties are increasingly being used to unravel complex biological mechanisms by exploiting InDels using resequencing analysis (Chung et al., 2014; Li et al., 2014; Song et al., 2015). Despite their biological importance, the investigation of InDels using a re-sequencing strategy has been limited. This requires the careful designing of InDel markers with proven quality and reproducibility which allows for constructing databases in order to share public use.

Soybean chromosomes are consisted of reshuffled sequence blocks that are originated from a limited number of ancestral contributors and were introduced relatively recently over the course of the past several decades (Haun et al., 2011). Alongside the technical development of NGS, the recombination block-based analysis is emerging as an efficient approach for identifying soybean cultivars with high accuracy to detect genetic diversity. Recently, Kim et al. (2014) determined two types of blocks by comparison of the genome sequences of six soybean cultivars: The sparse variation blocks (sVBs), which are identical or nearly identical to the reference sequence, and the dense variation blocks (dVBs), which contain many variations. The sVBs showed 6.6 times higher recombination rates than those of the dVBs, which can lead to the conservation of dVBs. This feature is important for yielding reproducible genotype data among different laboratories and detection platforms as well as over time.

**Figure 1:**
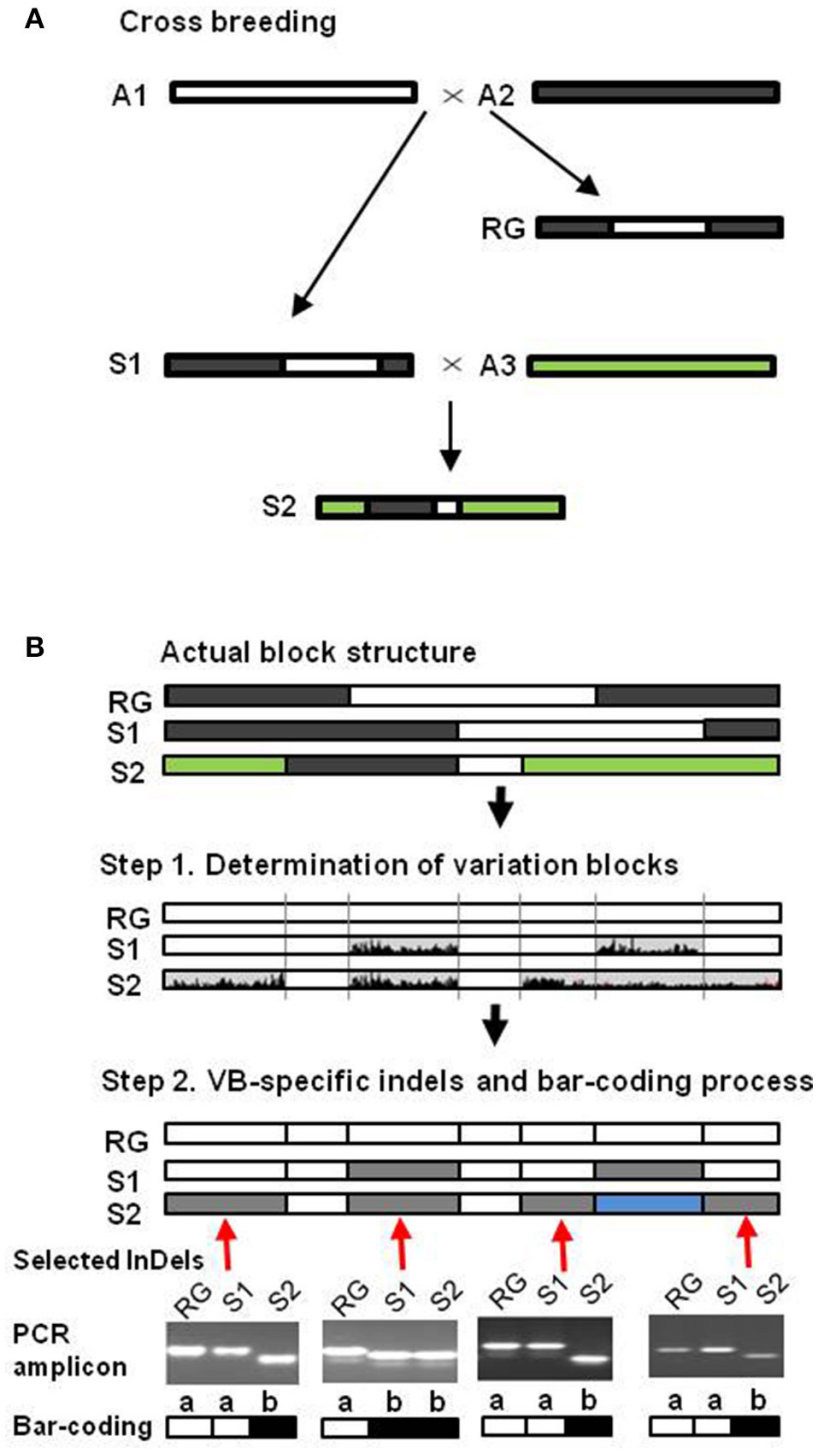
**Schematic representation of soybean barcode system using InDel markers. (A)** Sequence diversity generation by recombination events during the cross-breeding process. The rectangle represents the sequences, and the different colors indicate the divergence in the sequences. **(B)** Two steps were used for developing barcode system. In step 1, two types of blocks, the dense variation blocks (dVBs, gray) and the sparse variation blocks (sVBs, white), were determined by the sequence comparisons of cultivar genomes. In step 2, selection of dVB-specific InDels and bar-coding process. At each chromosomal position, dVBs of identical types were represented by the same color compared to reference genome. Based on the results of dVB-specific InDel marker amplification, the same results with the reference genome (RG) were represented by “a” (white), the other results by “b” (black).

The availability of a comprehensive set of resources including sequence data and dVBs facilitates the development of a soybean identification system using dVB-specific InDel markers (Kim et al., 2014). The key concept for establishing the soybean barcode system is to identify the block-based comparison with dVB-specific InDels that infers the homology of recombination blocks originated from the same parental genome. In this study, we developed soybean barcode system for the identification and breeding of varieties with minimal screening by using a small number of InDel markers, each of which is specific to a dVB. In order to develop such a soybean barcode system, three steps were used: In step 1, VB regions to provide reshuffling pattern of whole genomes were determined (Kim et al., 2014); in step 2, soybean barcode system for genetic identification were developed through selection of dVB-specific InDels and bar-coding process (Figure 1); finally, they were applied to the identification of 147 soybean cultivars for validation of the reliability of 202 InDel markers for discriminating soybean. Our approach provides significant information for not only the crop identification but also for breeding of varieties with minimal screening by using a small number of the selected InDel markers.

## Materials and methods

### Plant material and DNA extraction

Six commonly cultivated Korean soybean cultivars: Baekun (BU, IT142810); Sinpaldal2 (SP2, IT263155); Daepoong (DP, IT214690); Shingi (SG, IT214697); Hwangkeum (HK, IT157912); and Williams 82 (W82, IT163461) were used for exploiting dVBs and dVB-specific InDels. “DP” and “SG” were bred through a crossing combination of “BK” × “SP2.” “HK” is not a member of this family but is popular for its attractive color and bean size. Analysis of genetic diversity for dVB-specific InDels in terms of genetic identification was carried out on 147 soybean cultivars (Table S1) which came from the collection of soybean varieties of Department of Southern Area Crop Science at National Institute of Crop Science. Using standard protocols, genomic DNA was extracted from frozen young leaves of a bulk of 10 plants (Reyes-Valdés et al., 2013) grown in pots as previously described by Rogers and Bendich (1994), and used for exploiting dVBs and testing the newly developed markers, respectively.

### Identification and validation of the polymorphic InDels

Access to pair-end sequence data for the five Korean soybean cultivars was kindly provided from Kim et al. (2014). The data consisted of 101 or 104-bp reads generated using the Illumina GAIIx or HiSeq 2000 sequencer. There were a total of 40~59 Gbp sequence, which is 41~60-fold coverage. Insert size was estimated by mapping the reads to the reference *G. max* 109 soybean reference genome (Schmutz et al., 2010) using the Burrows-Wheeler Aligner algorithm (bwa: Li and Durbin, 2010) ver 0.5.9 allowing two mismatches and two gaps. The aligned reads were realigned at InDel positions with the GATK InDelRealinger algorithm (McKenna et al., 2010) for enhancing the mapping quality. The base quality scores were recalibrated by using the GATK TableRecalibration algorithm. The allelic diversity of InDels with the e-PCR products in five soybean genomes was assessed by PIC, which was defined as PIC *i* = 1- $\overset{n}{\underset{j=1}{\sum }}p_{ij}^{2}$, where p_*ij*_ is the frequency of the *j*th pattern for the *i*th marker (Anderson et al., 1993).

The sequence data derived from five soybean cultivars were compared with the reference *G. max* cv. Williams 82 genome by a variation block method (Kim et al., 2014). In this step, we exploited the dVBs which represented recombination sites. The InDel markers described in this study were identified/generated from dVBs in the six soybean cultivars (BU, SPD2, SG, DP, HK, and W82). The primer pair to amplify each of the InDels selected above was designed by Primer3 software (http://primer3.sourceforge.net). The InDels of at least 5~20 bp in length in the dVB regions were identified and the primers were designed accordingly to match the characteristics of each InDel by using the primer3 software (http://frodo.wi.mit.edu/primer3/). For limitation of the number of candidates, we chose primer pairs that amplified PCR products 80~120 bp long (Table S2).

### PCR amplification and gel electrophoresis

The PCR analysis was performed using 10 μL reaction mixtures containing 20 ng of total genomic DNA, 2 pM of primer, and 5 μL of GoTaq Green Master Mix (Promega, madison, WI, USA). PCR was performed under conditions of 95°C for 5 min and subsequent 35 rounds of 94°C for 30 s, 45°C for 30 s, and 72°C for 30 s, using a Biometra T1 Thermocycler (Biometra, Goettingen, Germany). The PCR products were separated by electrophoresis in 3% gel of certified low range ultra-agarose (Bio-rad) followed by ethidium bromide staining.

### Selection and evaluation of 202 dVB-specific InDels for genetically identifying soybean cultivars

The 202 InDel markers were selected from the previously developed 73,327 according to their genotyping success and PCR band size. The discriminating power of the selected 202 InDel set for soybean cultivar identification was evaluated with the 147 soybean cultivars (Table S1). The homology of soybean cultivars was calculated after the PCR amplification of all 202 InDels. In addition, a phylogenetic tree for the 147 soybean cultivars was drawn based on the genotypes defined using the 202 InDels using the weighted neighbor-joining method with simple matching coefficients implemented in the cluster 3.0 (de Hoon et al., 2004; available at http://bonsai.hgc.jp/~mdehoon/software/cluster) and the DARwin software (Perrier and Jacquemoud, 2006; available at http://darwin.cirad.fr/darwin).

## Results

### Putative InDel markers representing dVBs

**Figure 2:**
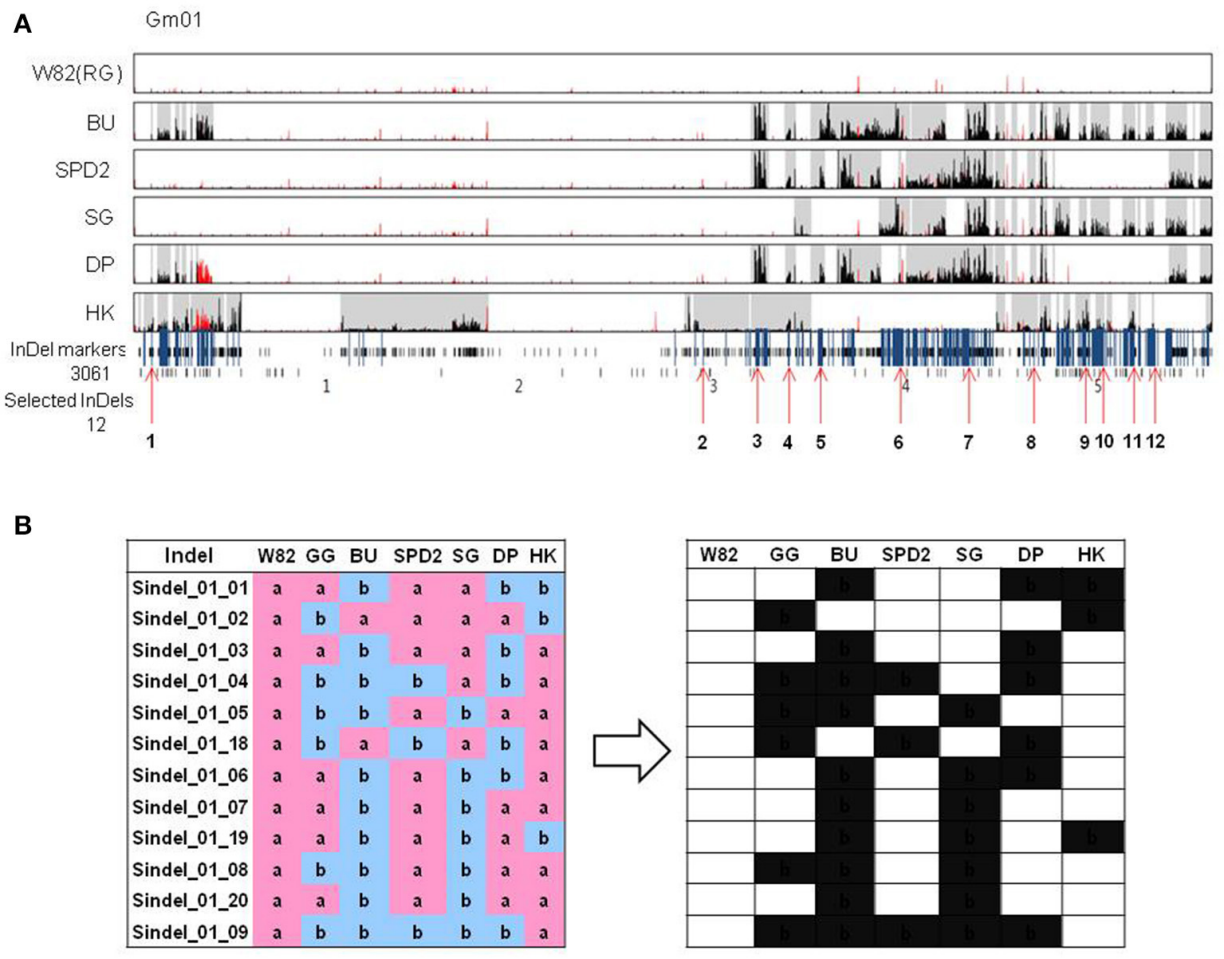
**Selection of InDel markers representing chromosomal variation and bar-coding process by using the selected InDels. (A)** Chromosomal variations and 12 InDel makers (red arrows) determined in chromosome 1 of the six cultivars. Five Korean soybeans were used to determine the variation block type and InDel markers. **(B)** Bar-coding representation of the polymorphisms revealed by the InDel markers among seven soybean cultivars. The same result with the genome of reference cultivar, Williams 82 is represented by “a” (pink), the other by “b” (blue). For barcode of the results, “a” and “b” were converted to white and black, respectively. W82, Williams 82; GG, Gwanggyeo; BU, Baekun; SPD2, Sinpaldal2; SG, Shingi; DP, Daepoong; HK, Hwangkeum.

Kim et al. (2014) sequenced whole genomes of six soybean cultivars including parental cultivars (Backun, BK and Sinpaldal 2, SPD2), their crossed descendants (Daepoong, DP and Shingi, SG), an elite cultivar (Hwangkeum, HK), and a reference (Williams 82, W82). To design InDel markers for discriminating soybean cultivars, sVBs and dVBs in accordance with the VB-based method in Kim et al. (2014), were selected from five soybean cultivar genomes and compared to the reference *G. max* cv. W82 genome. Especially, the dVBs were well-conserved due to lower recombination rates compared to the sVBs in the five soybean genomes. By comparing the dVBs among the genomes for identifying soybean cultivars, two dVBs with ≥99.8% sequence identity as well as ≥0.8 SNV concordance were considered to be of an identical type which were originated from a common parental genome (Kim et al., 2014). In chromosome 1 of six soybean cultivars, this permitted the identification of 112 dVBs, which itself allowed for selecting 3,061 putative InDels. These InDels were further reduced to 12 dVB-specific loci based on examination of the two-type band of PCR products (Figure 2). In addition, a total of 2,274 dVBs and 73,327 InDels were identified from the six soybean cultivars and these InDels were selected in order to compare with the reference genome to discriminate all genome types in soybean (Table 1).

**Table 1:** **Number of dense mutation block, designed, tested, and selected indel marker in each soybean chromosome**.

**No. of chromosome**	**No. of dense variation block**	**No. of designed InDel marker**	**No. of tested InDel marker**	**No. of selected InDel marker**	PIC^*^ value of InDel marker
Gm01	112	3,061	20	12	0.39
Gm02	123	3,466	20	12	0.40
Gm03	161	4,744	20	12	0.42
Gm04	113	3,336	20	11	0.42
Gm05	106	2,939	20	12	0.41
Gm06	121	3,525	20	9	0.41
Gm07	144	3,444	20	8	0.43
Gm08	126	3,047	20	10	0.42
Gm09	124	4,430	20	11	0.36
Gm10	136	2,979	20	9	0.36
Gm11	111	2,126	20	10	0.36
Gm12	119	2,781	20	8	0.38
Gm13	111	4,102	20	9	0.41
Gm14	65	4,217	20	11	0.30
Gm15	84	4,572	20	10	0.39
Gm16	73	4,281	20	8	0.39
Gm17	116	3,161	20	12	0.37
Gm18	100	6,751	20	10	0.36
Gm19	118	3,894	20	10	0.34
Gm20	111	2,471	20	8	0.36
Total	2,274	73,327	400	202	0.38

* *PIC means Polymorphism Information Content*.

### Selection of 202 dVB-specific InDels and bar-coding process

Sequence comparison at nucleotide level of the tested genomes with the reference genome revealed PCR-based InDels. To facilitate screening using gel-electrophoresis, only InDels of 5~20 bp in length were selected and converted them as PCR-based markers. We tested the 400 primer sets, focusing on their ability to amplify PCR products in the six genome types, since this study aimed to establish PCR-based markers applicable for all soybean varieties. In the second assessment, the 202 InDels that gave clear PCR bands (80~120 bp) in the six soybean cultivars were selected and further analyzed. The 202 InDel loci were widely distributed on whole 20 chromosomes in the six soybean cultivars (Figure S1). Moreover, for the selected 202 InDel markers, the average PIC value was 0.38 with a range of 0.30–0.43, which implied that the selected InDels could be applied for investigating polymorphisms of soybean cultivars (Table 1).

**Figure 3:**
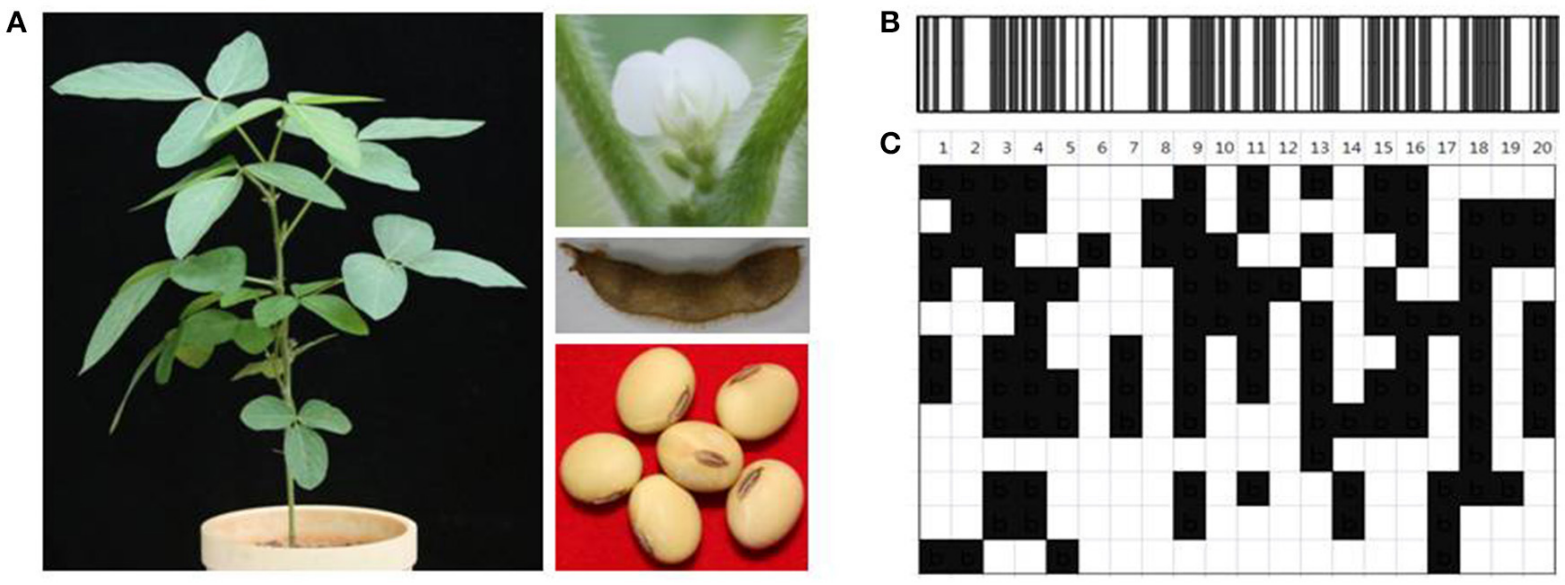
**Application of soybean barcode system to soybean cultivar, Daepoong. (A)** Phenotype of soybean cultivar, Daepoong. PCR results using 202 InDels in “DP” were converted to **(B)** standard 1D and **(C)** widely used 2D barcode types in comparison with those in “Williams 82.” The same results with reference genome Williams 82 were represented by white, and the other results by black. The upper lines show chromosomal numbers.

Primers were designed by targeting the InDel region, in such a way that the genotypes of soybean cultivars would produce same or different (insertion or deletion) amplicons relative to reference genome (Table S2). Based on the results of InDel marker amplification, the same results with “W82” were represented by “a”, the other results by “b”, which were depicted as “white” and “black” barcode, respectively (Figure 2). Figure 3 shows that the soybean barcode system actually was applied to soybean cultivar “DP” in respect to the reference. The PCR results using 202 InDels in “DP” were converted to standard 1D and widely used 2D barcode types according to comparing amplicon polymorphisms in soybean cultivar “DP” to “W82.” The soybean barcode system with the selected 202 InDel set was built, and their stability and quality for genetic identification was thoroughly evaluated.

### Evaluation of the selected InDels for genetic identification

**Figure 4:**
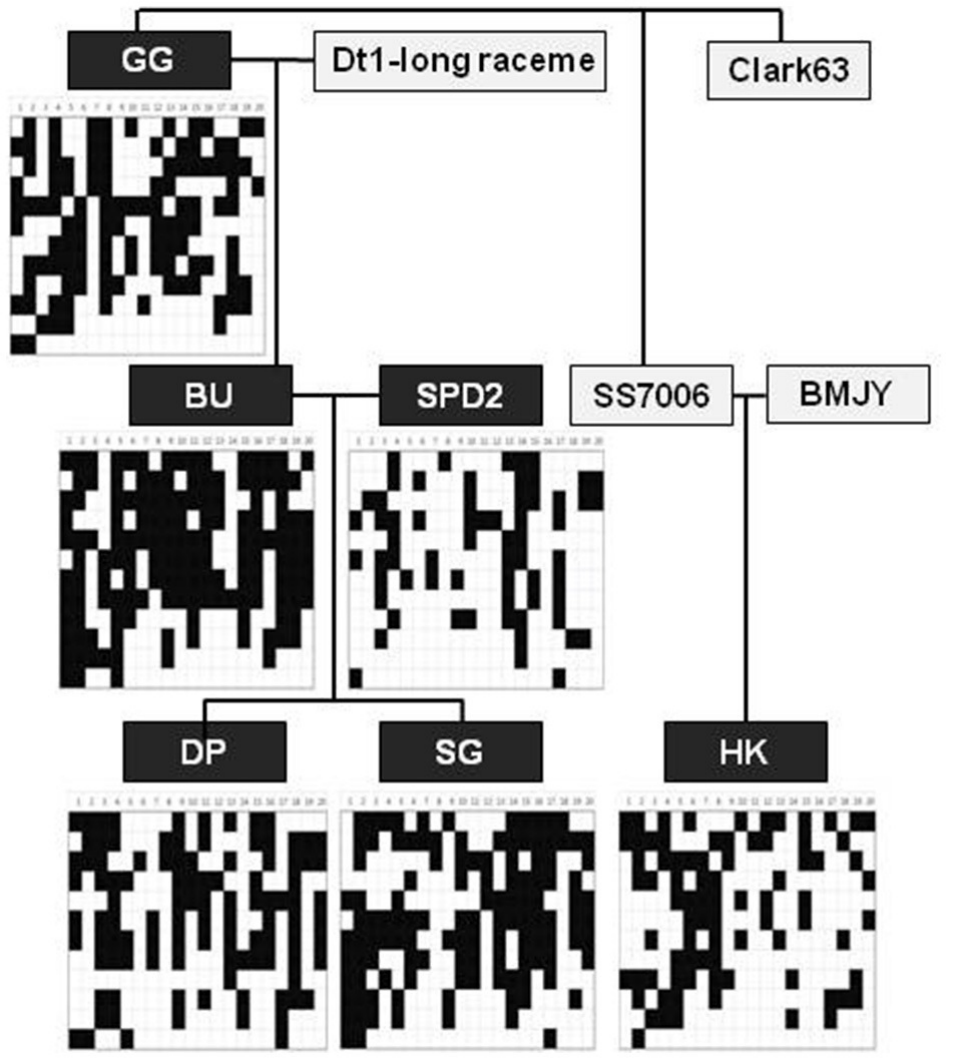
**Application of soybean barcode system to pedigree analysis in whole chromosomes of six soybean cultivars**. After PCR amplification using 202 InDel markers in seven soybean cultivars, the same results with the genome of reference cultivar, Williams 82 were represented by white, the other results by black. GG, Gwanggyeo; BU, Baekun; SPD2, Sinpaldal2; DP, Daepoong; SG, Shingi; HK, Hwangkeum; BMJY, Baekmokjangyeop.

The soybean barcode system was evaluated through the analysis of the genotypes obtained for six cultivars. These genotyping allowed for pedigree analysis with six cultivars, which represent a larger phenotypic diversity for important traits including yield stability (low, medium, and high), hilum color (brown and yellow), and other traits. To access the values of the InDel markers for pedigree analysis, parental cultivars (“BU” and “SPD2”), their crossed descendants (“DP” and “SG”), and an elite cultivar (“HK”) were selected to show dVB patterns on whole genomes. Figure 4 shows comparison of barcode types between two descendants (“DP” and “SG”) and its parents by using soybean barcode system. Almost all of dVB types in the descendants were present in the corresponding parental cultivars. However, few of dVB types (~1%), such as InDel_01_04 in chromosome 1 of “SG,” were not observed similarly in the parental genomes, likely because the two individual parental plants that were used in this analysis are not the direct ancestors of the descendant cultivars.

**Figure 5:**
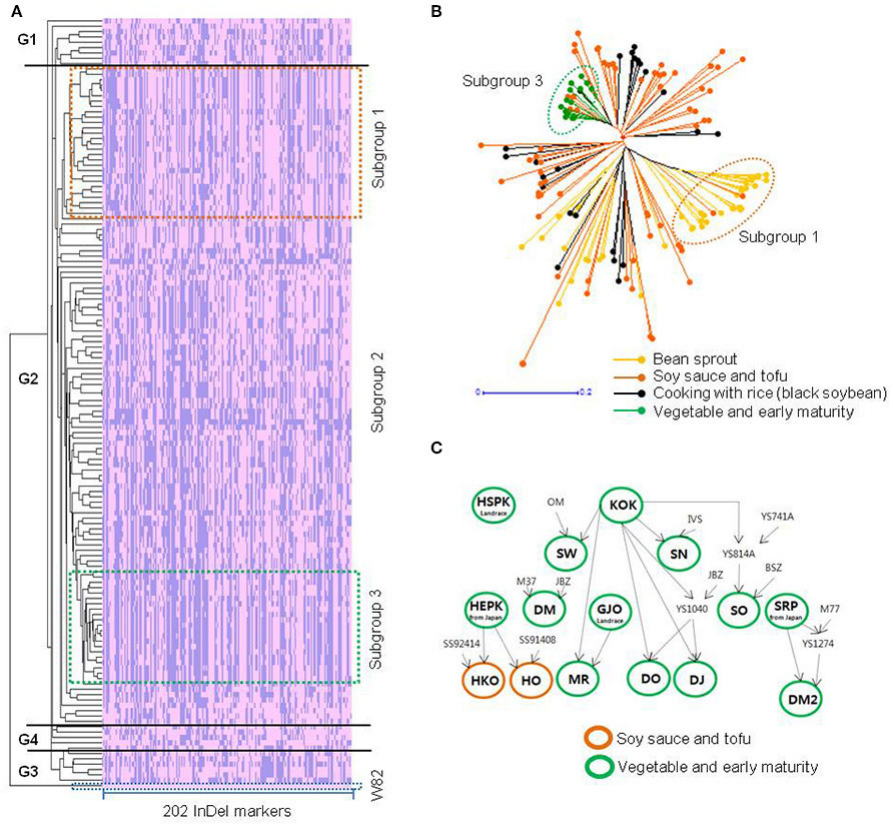
**Relationships among the soybean cultivars by using weighted neighbor-joining analysis of 202 InDel markers and pedigree analysis. (A)** Phylogenetic relationships with variation blocks of 147 soybean cultivars with cluster 3.0 software. Pink bars represent the same results with the reference genome, Williams 82 (the last line); blue bars represent the different size with Williams 82. W82, Williams 82. **(B)** Phylogenetic analysis among the 147 soybean cultivars with simple matching coefficients implemented in DARwin software. The horizontal bar indicates distance based on the simple matching coefficient. **(C)** Pedigree analysis of subgroup 2. HSPK, Hwaseongputkong; KOK, Kunolkong; SW, Sangwon; SN, Seonnok; HEPK, Hwaeomputkong; DM, Danmi; GJO, Geomjeongol; SO, Saeol; SRP, Seokryangputkong; HKO, Hwangkeumol; HO, Hanol; MR, Mirang; DO, Daol; DJ, Dajin; DM2, Danmi 2.

To validate the reliability of the 202 InDel markers for discriminating soybean varieties, bin maps with the InDels were constructed for 147 soybean cultivars. Weighted neighbor-joining (NJ tree) relationship with the bin maps revealed four groups (G1, G2, G3, and G4) that contained 9, 127, 7, and 4 accessions, respectively. Among G2, the NJ tree analysis also clustered the soybean cultivars into three subgroups by use in Korea (Figure 5). The color-coded branches supported the three subgroup classification. Subgroup 1 mainly consisted of cultivars for bean sprouts (92.9%), subgroup 2 comprised cultivars for soy sauce and tofu and cooking with rice (90.8%), and subgroup 3 consisted of cultivars for vegetable and early maturity (75.0%). Modern improved cultivars for vegetable usage have been selected to have both early maturity and large seeds. In contrast to vegetable soybean, small seeds in bean sprouts were preferred for the high yield of sprouting. It is likely that specific dVBs with extremely reduced diversity might be associated with such traits. Therefore, their clustering is strongly influenced by the difference in breeding ancestors among the subgroups (Figure 5), which resulted in the change in the reshuffling patterns of the soybean cultivars. Soybean chromosomes in descendants are all determined by genetic reshuffling of dVBs inherited from parental chromosomes. Thus, the reshuffling patterns of dVBs can explain genetic difference of the 147 soybean cultivars and how dVBs are inherited from ancestor chromosomes. This indicates that the developed InDel markers are very useful for genetic identification by analyzing the reshuffling patterns of the parental genomes in the descendants.

### Constructing database obtained using InDels in 147 soybean cultivars

**Figure 6:**
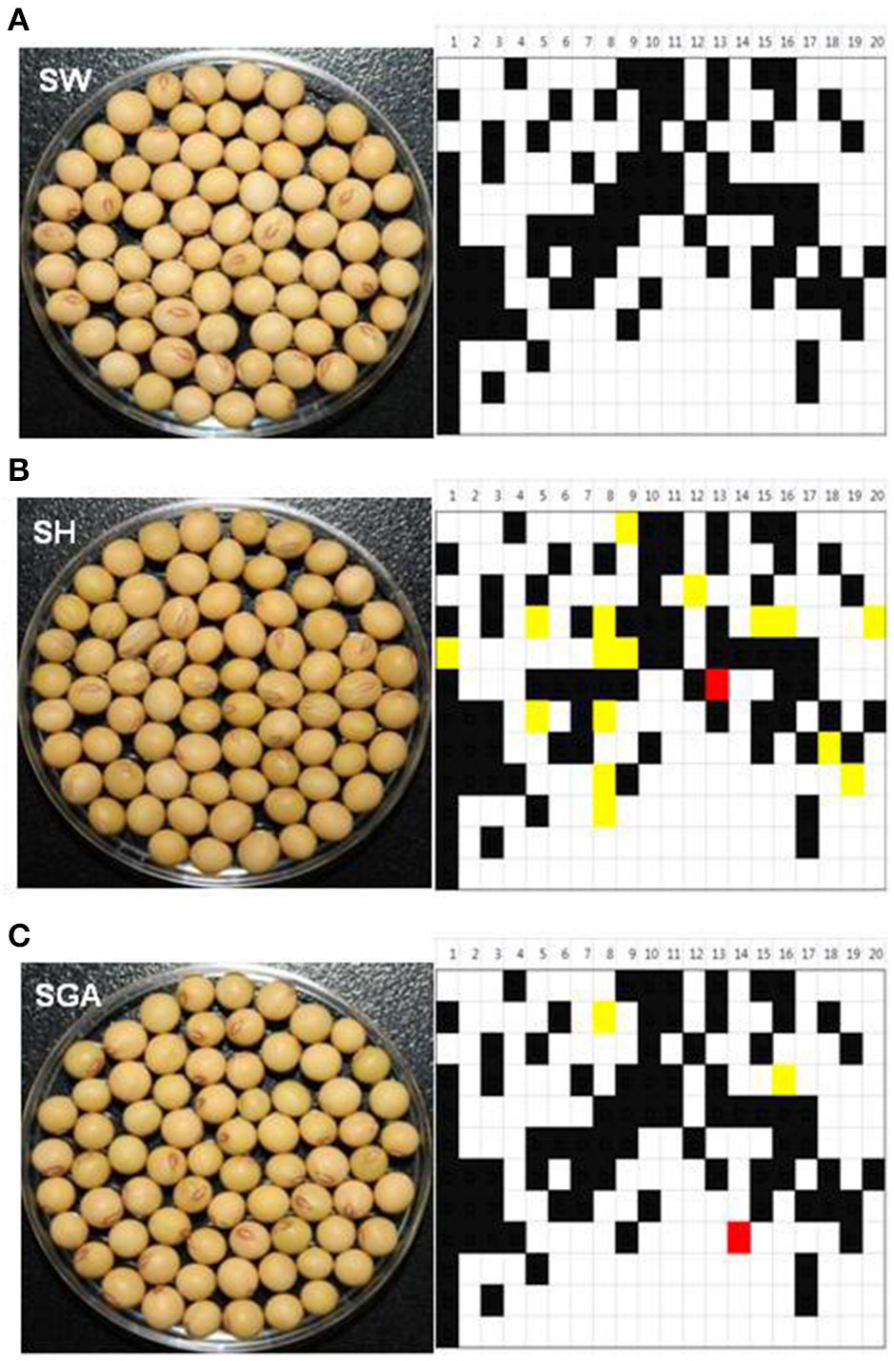
**Application of soybean barcode system to backcross selection**. After PCR amplification using 202 InDel markers in **(A)** a recurrent parent, SW/backcross-inbred off-springs, **(B)** SH, and **(C)** SGA, the same results with the genome of reference cultivar, Williams 82 were represented by white, the other results by black. Yellow shows heterozygous region. Red indicates target locus, *Rsv1* in SH and *Rsv3* in SGA. SW, Sowon; SH, Sinhwa; SGA, Singang.

By building database of the 202 InDel polymorphisms in the 147 soybean cultivars, we have established more stable foundation for utilizing soybean barcode system to provide a promising tool for soybean identification. The average difference between analyzed cultivars was 88 InDels from a total of the 202 InDels while the most different cultivar, BU differed in the 146 InDels compared to a reference genome, “W82.” The closest cultivars were “Singang (SGA)” and “Sowon2010 (SW2010),” which differed in three and four InDels out of the 202 when compared with “Sowon (SW)” (Figure 6). These cultivars have genotypes that are compatible with being backcross-inbred off-springs (“SGA” and “SW2010”)/a recurrent parent (RP, “SW”), based on dVB-specific InDel markers used in this study. The next closest cultivar, Sinhwa (SH) has been described as a sibling of the following cross: “PI96983” × “SW” (RP). The same result has been predicted in “SH”, which matched for 185 InDels. As shown in Figure 6, the genetic difference among the varieties with high genetic similarities was determined through their dVB comparison and measured by the reshuffling pattern of dVBs. Hence, it clearly implied that, even though the cultivars are genetically close, a varied difference has been measured in the number of diverse InDels.

**Figure 7:**
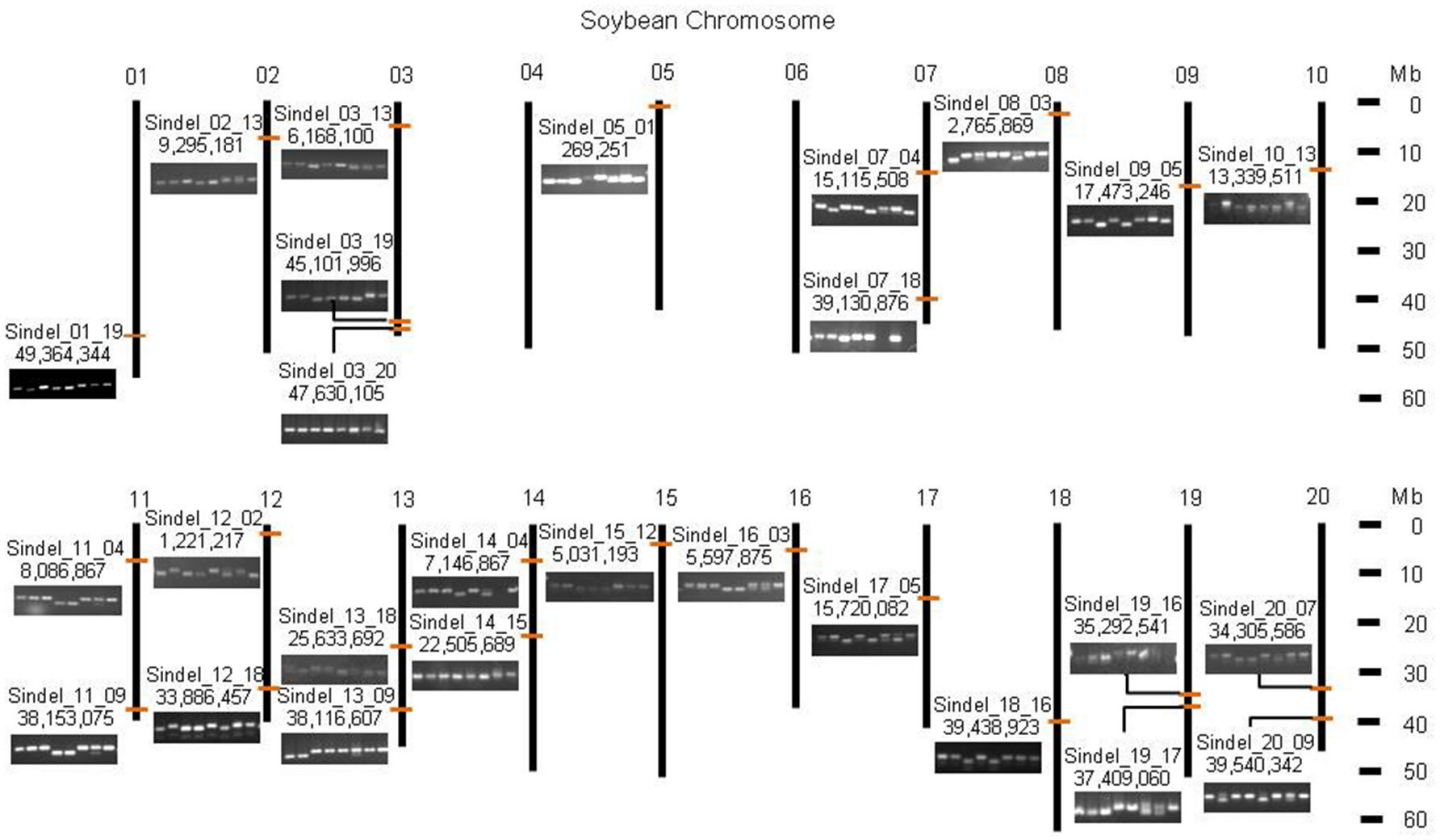
**The 27 InDel markers developed to discriminate 147 soybean cultivars**. The 27 evenly distributed InDel markers were recommended for genetic identification. Related primer sequences were represented on Table S2. The orange bars mean the 27 InDel markers selected in soybean chromosomes.

Among the 202 InDels, 27 markers with the comparison of 2-D barcode patterns in the 147 soybean cultivars were selected and subjected to cultivar identification analysis. In the 147 genotypes using 27 InDels, there was a very clear distinction between the closest varietal variability with 1 different allele and the most different varietal distance with 21 different alleles. For the InDel markers, the average PIC value was 0.37 with a range of 0.05~0.50. Among them, the least polymorphic marker was Sindel 18–16 which only showed in “BU,” “DP,” “Muhankong,” and “Socheong 2,” while Sindel 3–20 showed polymorphism in 76 of the 147 soybean cultivars (Figure S2). Moreover, these 27 markers are distributed evenly on whole chromosomes and produce PCR bands using normal PCR and electrophoresis conditions, very suitable for genetic identification (Figure 7). Thus, there should not be any difficulty in identifying the 147 soybean cultivars using the 27 InDels and it is surplus as to be considered conclusive for establishing distinctness in 147 soybean cultivars. Through investigation of the reshuffling pattern of new varieties with the 27 maker sets, the changing of dVBs in a chromosomal level can be quickly identified.

### Application of soybean barcode system to various breeding processes

**Figure 8:**
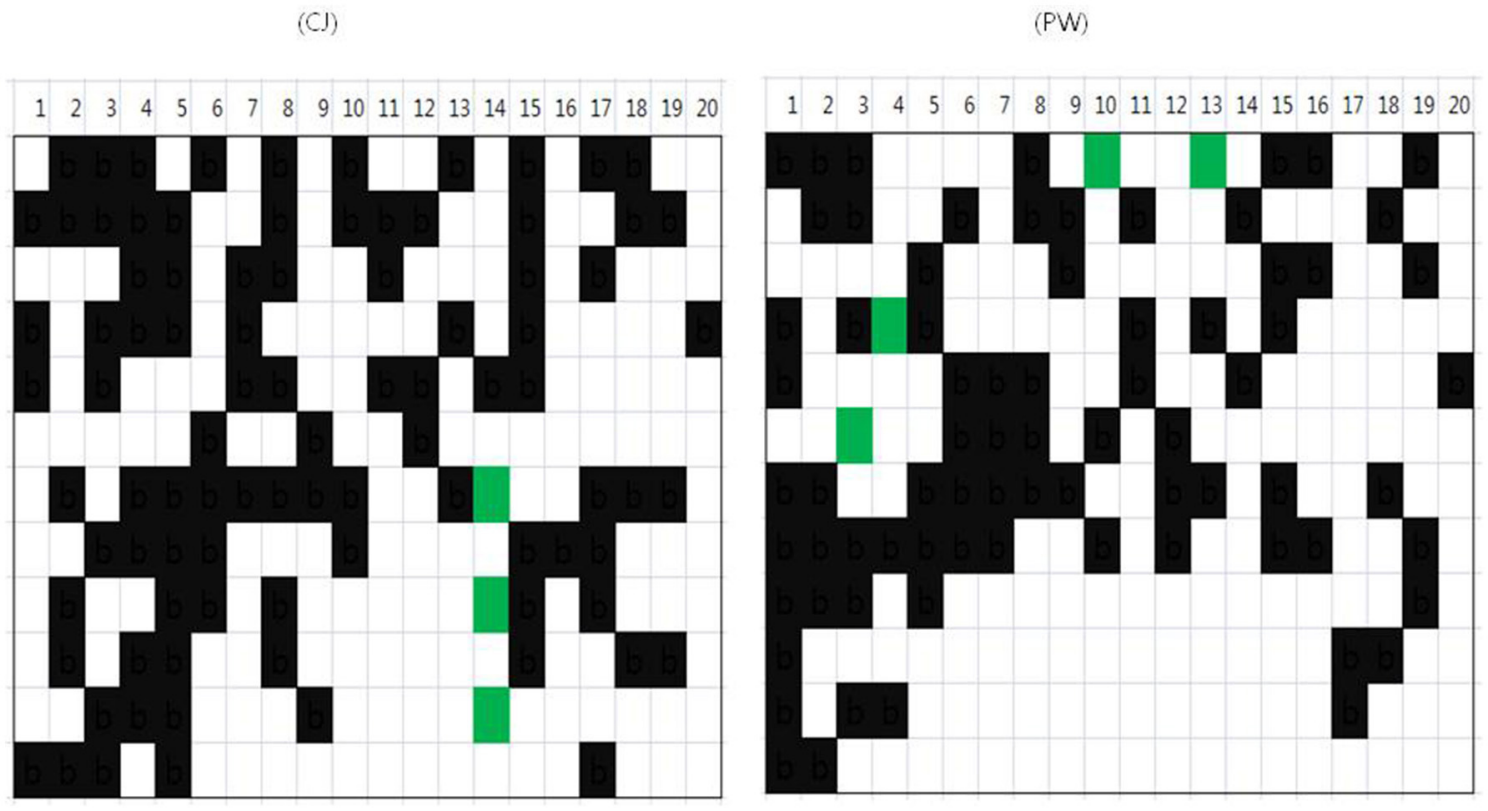
**The study of fixation rate in cultivars by using soybean barcode system**. The same results with reference genome Williams 82 were represented by white, and the other results by black. Green means heterozygous type. The upper lines show chromosomal numbers. CJ, Cheongja; PW, Pungwon.

As represented in Figure 6, the process of introgression of genes and recovery of the RP genome could be accelerated by selection using dVB-specific InDels. We showed the practicability of the soybean barcode system, which is useful for breeding varieties with minimal screening by analyzing the reshuffling patterns of soybean varieties. In the case of crop varieties developed by cross-breeding methods, the fixation of breeding varieties is critical to the uniformity and stability of varieties. When the soybean barcode system was applied to the 147 soybean cultivars, Cheongjakong (three dVBs in Gm14) and Pungwonkong (each dVB in Gm03, 04, 10, and 13) showed green blocks as heterozygous type indicating not to be completely fixed (Figure 8). This result highlights that the soybean barcode system can be effectively used to investigate the degree of fixation of soybean varieties. In addition, the soybean barcode system is effective for the selection of pure lines due to an exploration of whether areas of soybean chromosomes are heterozygous or not. These results show the practicability of the soybean barcode system, which is useful for breeding varieties with minimal screening by analyzing the reshuffling patterns of soybean varieties.

## Discussion

The discovery of a large number of genome-wide SNPs using next-generation sequencing technology has helped researchers to genetically characterize the soybean genomes at extremely high resolution (Kim et al., 2014; Lee et al., 2015; Shi et al., 2015). It is believed that the genetic differences of soybean cultivars can be represented by recombination blocks derived from the comparison of these SNP density profile in whole genomic regions of the six soybean cultivars (Kim et al., 2014). In the present study, we propose an efficient genetic identification method that is based on InDel markers, each of which is specific to a recombination block originated from parental genomes.

### The selection of dVB-specific InDels for genetically identifying the soybean cultivars

In general, artificial selection results in new polymorphisms generated by three processes: The appearance of new SNPs, the recombination of existing genome segments, and a combinations of these two processes (Hyten et al., 2006; Stefaniak et al., 2006; Yonemaru et al., 2012; Kim et al., 2014). In Korea, elite varieties were introduced and used as donors for breeding soybean cultivars to maintain favorable phenotypes such as eating quality (Kim et al., 2012). Our phylogenetic and pedigree analysis in soybean cultivars also supports our belief that modern Korean soybean cultivars were mainly derived from crosses among a limited number of elite varieties (Figure 5). This observation is consistent with previous study that soybean cultivars for vegetable and early maturity showed the lowest genetic diversity (Kim et al., 2006). In addition, few mutation has been accumulated for the short modern of Korean soybean breeding. The available evidence therefore suggests that soybean cultivars have low levels of genetic diversity but were derived from genetically reshuffled recombination blocks of breeding parents (Hyten et al., 2006; Stefaniak et al., 2006).

To identify soybean cultivars with high accuracy, VBs should be conserved during soybean breeding, and VBs as recombination blocks have been reported for soybeans (Hyten et al., 2007; Kim et al., 2014). Kim et al. (2014) showed that the genetic inheritance of dVB-specific InDel markers has been confirmed in previously described 614 F4 progenies of recombination inbred lines that were selected from the cross of “HK” and “DP”. Linkage disequilibrium (90~574 kb) in three cultivated *G. max* groups (Hyten et al., 2007) supports our belief that the dVBs (<100 kb) mainly in gene-rich region should be much conserved during breeding process. In this study, as shown in Figures 4, 5, the pedigree and the NJ-tree analysis showed the conservation of dVBs after repeated propagation of the varieties. There were identical variation patterns consistently appearing in the same dVBs of the examined InDels, indicating that the dVBs were inherited from common ancestors. In particular, the dVBs arising from a limited number of parental varieties used during modern selection breeding in Korea should facilitate a block-based comparison for soybean identification.

For soybean cultivar identification, we analyzed the 202 dVB-specific InDels which were selected from the whole genome of the six soybean cultivars using a re-sequencing strategy. These InDels that gave clear PCR bands (80~120 bp) were widely distributed on whole chromosomes in the six cultivars (Table 1 and Figure S1). The genetic inheritance of the loci has been confirmed in pedigree analysis (Figure 4). Moreover, in 147 soybean cultivars, most of the InDels (95%, 199/202) produced two allele products, and the remaining three markers detected a third allele in one to four cultivars respectively (Figure 5). Especially, backcross-inbred off-springs (“SGA” and “SW2010”) and an RP (“SW”) were distinguished by using these InDel markers (Figure 6). Especially, the 202 dVB-specific InDels should be stable, meaning that they produce consistent and reproducible genotype data among different laboratories and detection platforms as well as over time. This feature is especially useful in genomic identification of soybean cultivars and allows for construction of a publicly available genotype database and direct comparison of data arising from different sources.

### Soybean barcode system using the dVB-specific InDel markers

To efficiently identify soybean cultivars, we developed the barcode system using the 202 dVB-specific InDels which were selected from the genome of the six soybean cultivars using a re-sequencing strategy. The VB-based soybean barcode system has several advantages over other genetic identification methods. The first advantage is that the system is efficient, rapid and cheap for genetic identification of soybean cultivars in common laboratories. The system can easily identify soybean cultivars by using PCR and gel-electrophoresis based apparatus compared to other molecular markers, such as SSR and SNP (Hou et al., 2010; Mullaney et al., 2010; Pacurar et al., 2012; Montgomery et al., 2013; Yamaki et al., 2013; Moghaddam et al., 2014; Wu et al., 2014). Moreover, the availability of a comprehensive set of resources including sequence data and dVBs make it easier to develop a platform using dVB-specific InDel markers (Kim et al., 2014).

The second advantage is that the soybean barcode system does not depend on the number of samples. Each soybean variety shows a unique dVB pattern which can be distinguished from other varieties (Kim et al., 2014). This fact was confirmed by constructing the database of the 147 soybean cultivars, indicating that the 202 dVB-specific InDels should be widely transferable and reproducible for genetically identifying soybean cultivars (Figures 5, 6). Thus, the reshuffling patterns of new cultivars can be investigated by using the 202 dVB-specific InDels without developing additional markers in new cultivars.

The third advantage is that the barcode system can accurately recognize the difference of 2-D barcode pattern in soybean cultivars for easier visual identification. There is no need to use InDel markers on the dVBs of the same type that are present in two genomes due to direct comparison of the cultivars. In this study, through the 2-D barcode pattern comparison using the dVB-specific InDel markers, we have already demonstrated this by identifying the 147 soybean varieties with the 27 InDel markers, indicating that the 27 InDels should be proposed as a minimum set for genetic identification (Figure 7). Therefore, soybean cultivars can be identified with reduced screening efforts by using a small number of InDel markers that represent the dVBs.

In the barcode system, highly accurate identification relies on a large scale genotyping which increases the cost of constructing database. A number of recent papers have proposed bulk DNA sampling for germplasm characterization as a remedy for this (Michelmore et al., 1991; Dubreuil et al., 1999; Sham et al., 2002). In this study, a bulk of 10 plants was used to construct genotyping database of the 147 soybean cultivars through InDels with a good control of the dilution problem. DNA pooling is useful in constructing database through large scale genotyping to reduce the cost of analyzing genetic markers for the barcode system (Reyes-Valdés et al., 2013).

### Further studies for application of the soybean barcode system

In the future, the genotyping using the barcode system will be connected with phenotypic information through comparison of dVBs in soybean cultivars. As presented in Figures 6, 8, the changing of dVBs in a chromosomal level can be quickly navigated due to investigation of the reshuffling pattern of soybean varieties. This feature is useful in furthering the understanding of the genetic architecture related to the valuable target traits. Actually two or more traits are often the targets of improvement in the development of plant varieties. Even, when only one trait is a target, it is necessary to evaluate the genetic potential of multiple traits that are agronomically important (Salome et al., 2011; Xu et al., 2013). In addition, InDels are increasingly being used to unravel complex biological mechanisms of diverse soybean cultivars (Chung et al., 2014; Li et al., 2014; Song et al., 2015). Therefore, considering the practicability of the system for map-based screening and soybean breeding, the system is of great value in common laboratories (Kim et al., 2014).

In summary, the barcode system has been developed with the 202 dVB-specific InDel markers selected through comparing the whole genomes of the six soybean cultivars and tested for genetic identification purposes. We demonstrated the usefulness, reliability and accuracy of the soybean barcode system by applying it the publicly available 147 soybean genomes for cultivar identification. The dVB-based barcode system does not require any allele binning and thus, the barcode system is suitable for the building of a publicly available genotype database for soybean cultivars. Therefore, we propose that the soybean barcode system using the dVB-specific InDel markers is useful for identification of soybean varieties. Further studies of comparison between phenotypes and dVB-based genotypes would be helpful for application of the system to marker-assisted soybean breeding in common laboratories.

## Author contributions

TH, DL, and SL performed the experiments. HS, SK, TH, SH, YS, BK, and YK analyzed the data. HS, SK, and KM drafted the manuscript. HP, YL, and YK designed the project. YK supervised the project and complemented the writing.

### Conflict of interest statement

The authors declare that the research was conducted in the absence of any commercial or financial relationships that could be construed as a potential conflict of interest.

## Supplementary material

Figure S1 **The position of 202 InDel markers in soybean genomes**.

Figure S2 **A diagram of PCR band patterns of 147 soybean cultivars obtained using 27 InDel markers**.

Table S1 **List of 148 soybean samples used for the validation of the soybean barcode system**.

Table S2 **The 202 InDel markers developed to discriminate soybean cultivars in this study**.
