# Supplementary material for: Barcode System for Genetic Identification of Soybean [Glycine max (L.) Merrill] Cultivars Using InDel Markers Specific to Dense Variation Blocks
Source: Front Plant Sci. 2017 Apr 10;8:520. doi: 10.3389/fpls.2017.00520 (PMC5385371; doi:10.3389/fpls.2017.00520)
Supplement: Figure S1 — The position of 202 InDel markers in soybean genomes. [file DataSheet1.pdf]

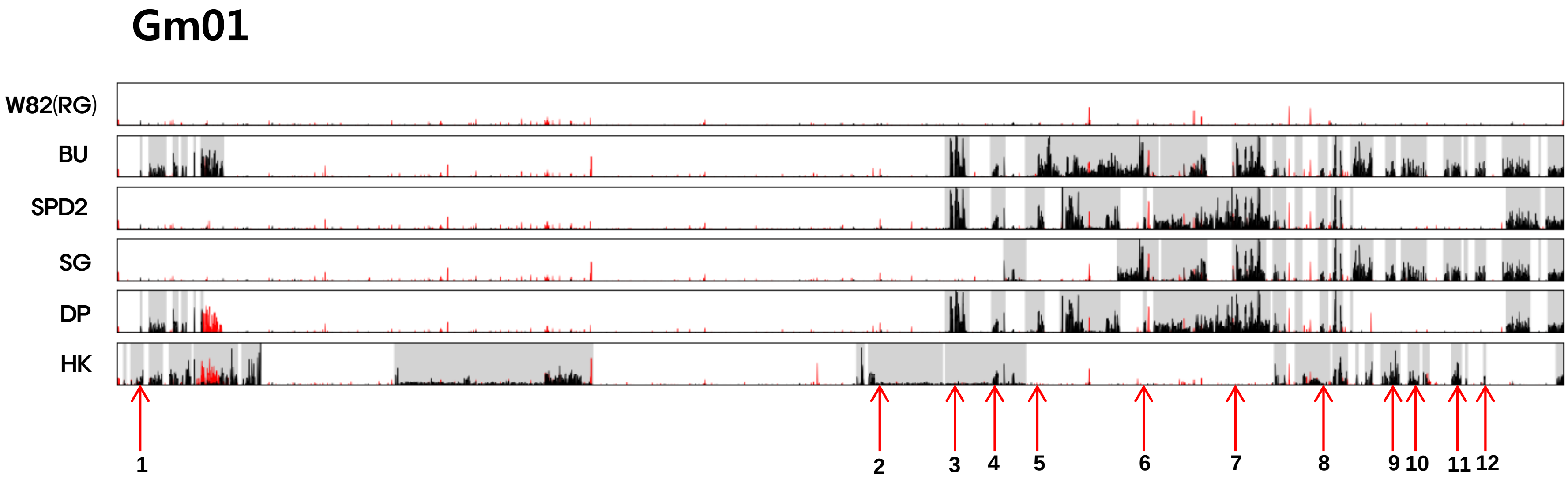
Gm01
W82(RG)
BU
SPD2
SG
DP
HK
1
2
3
4
5
6
7
8
9
10
11
12

Gm02

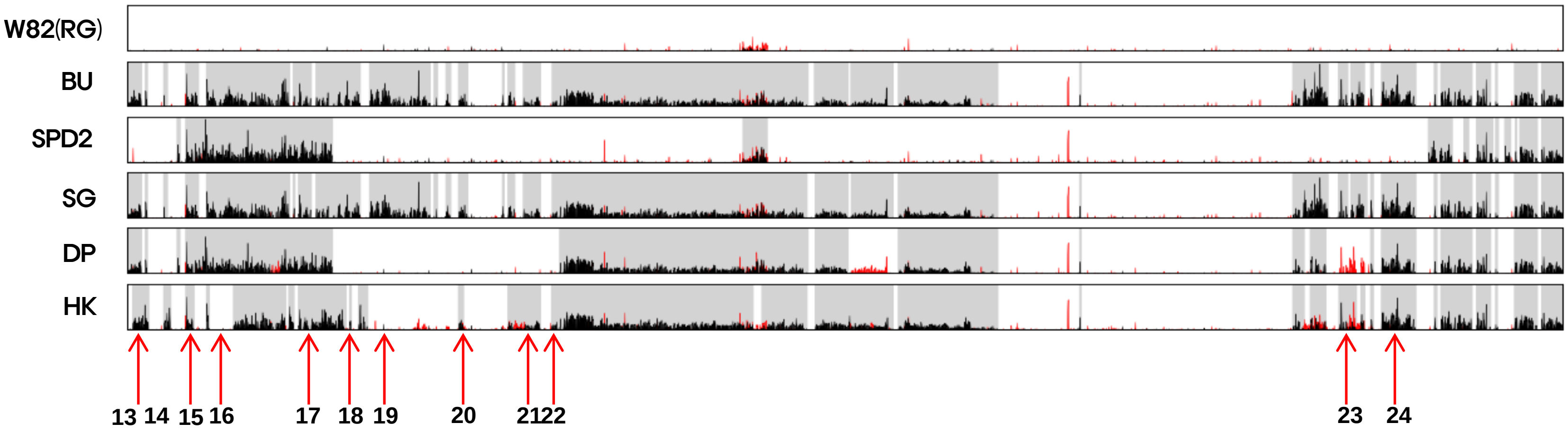

Figure S1. (Continued)

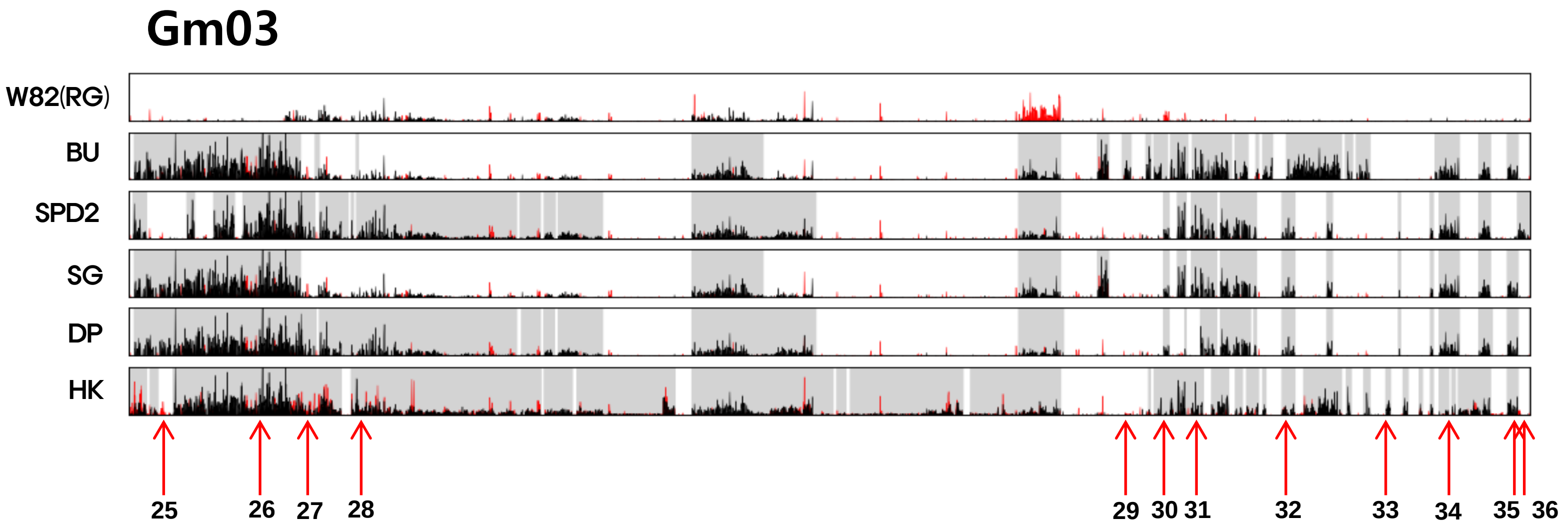

Figure S1. (Continued)

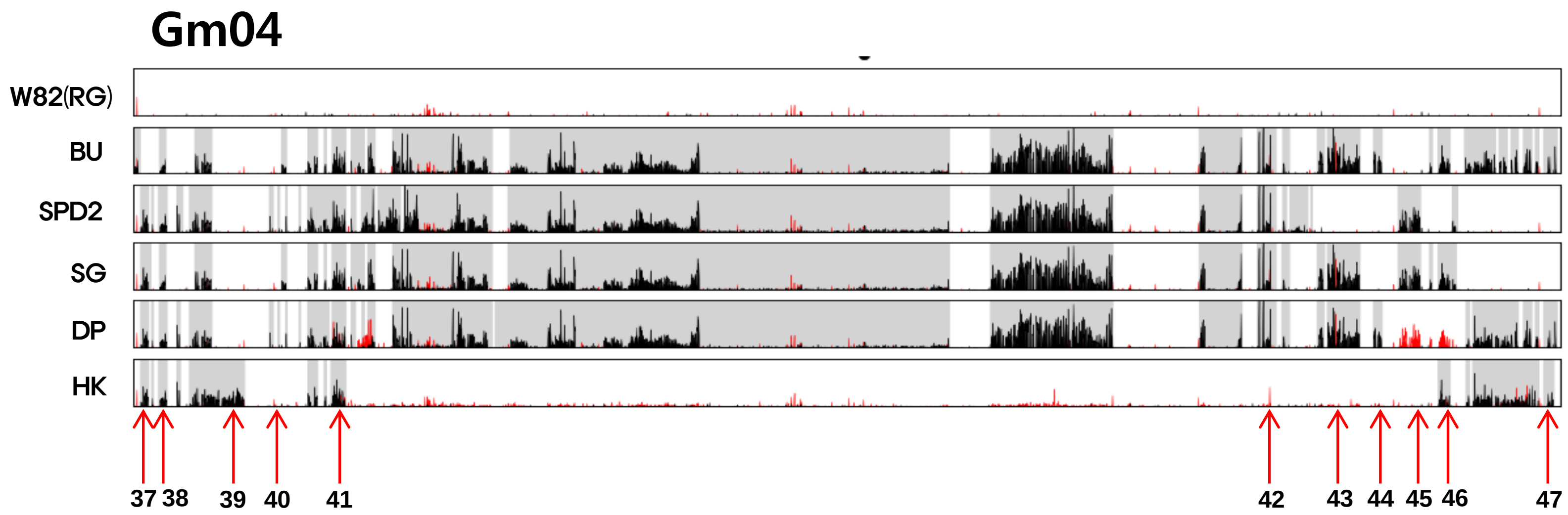

Figure S1. (Continued)

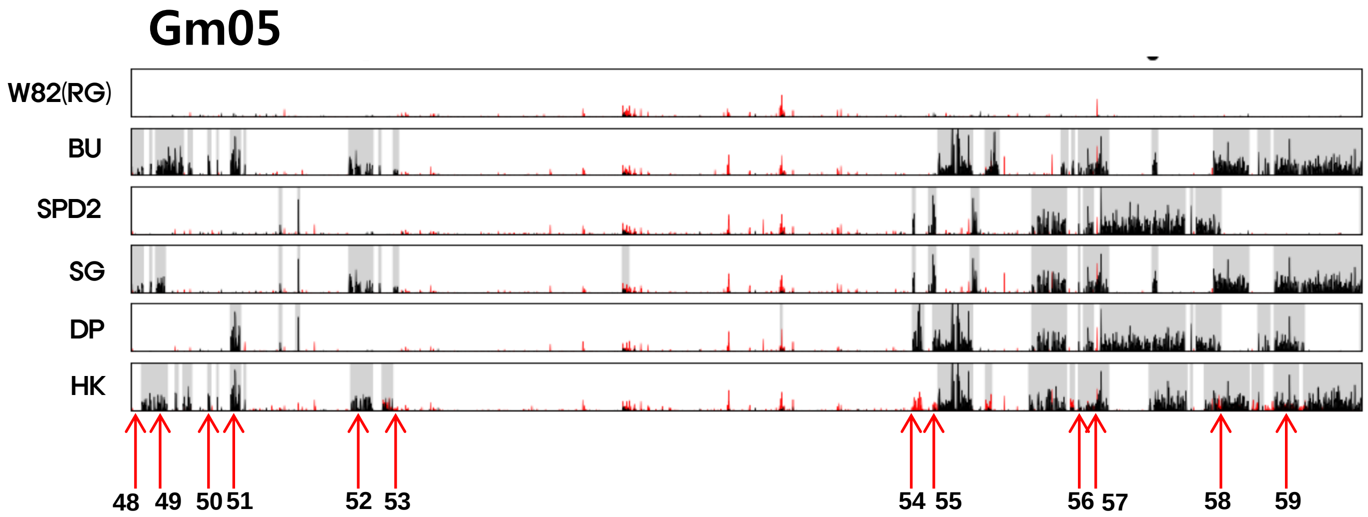

Figure S1. (Continued)

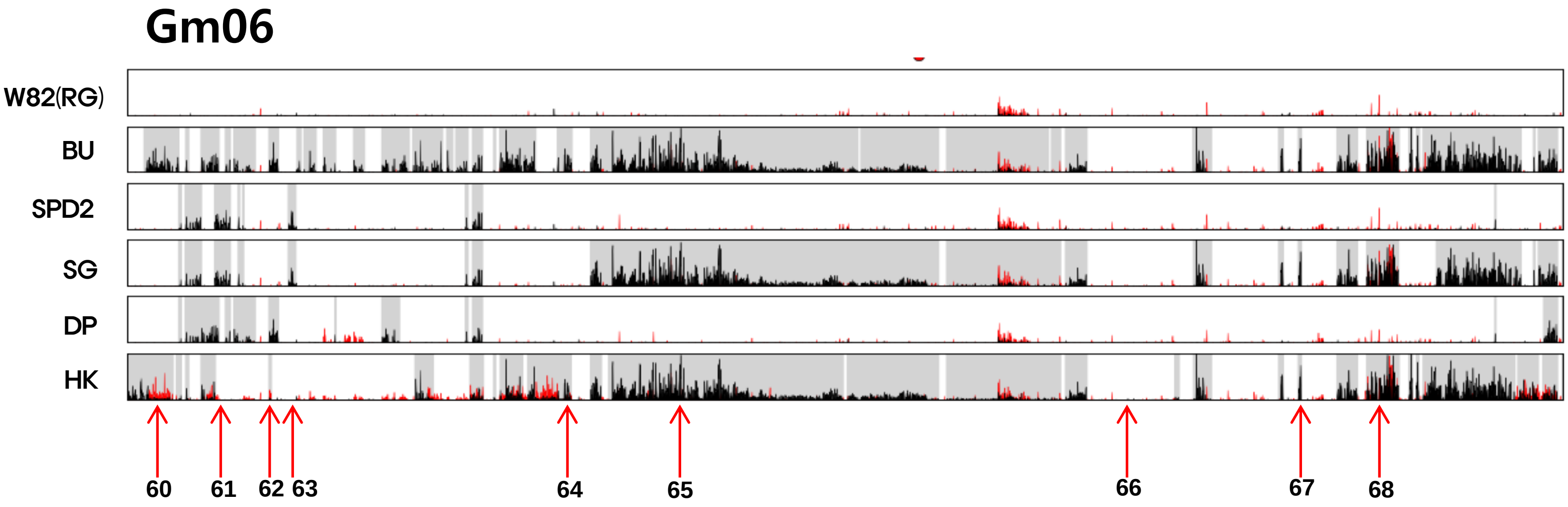

Figure S1. (Continued)

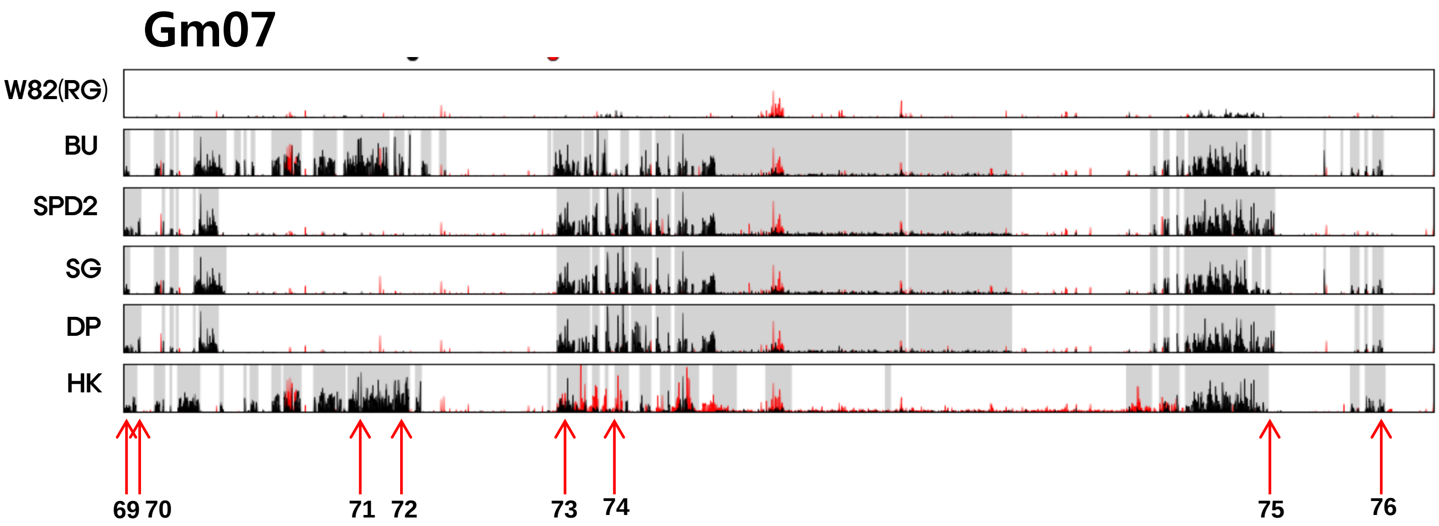

Figure S1. (Continued)

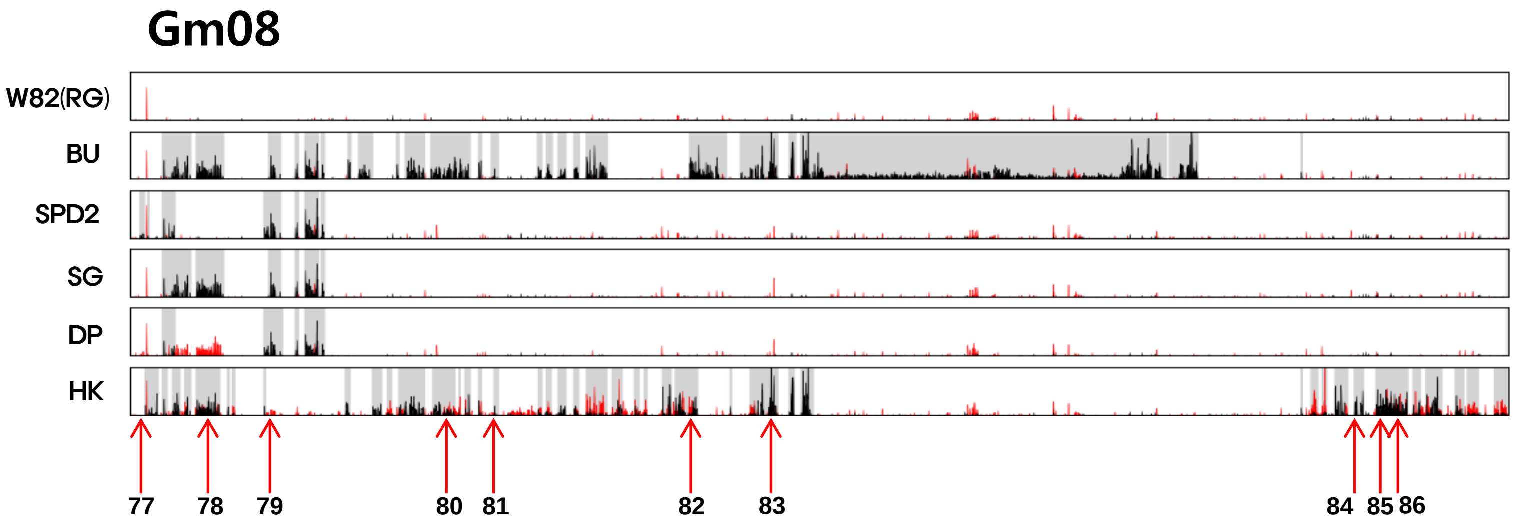

Figure S1. (Continued)

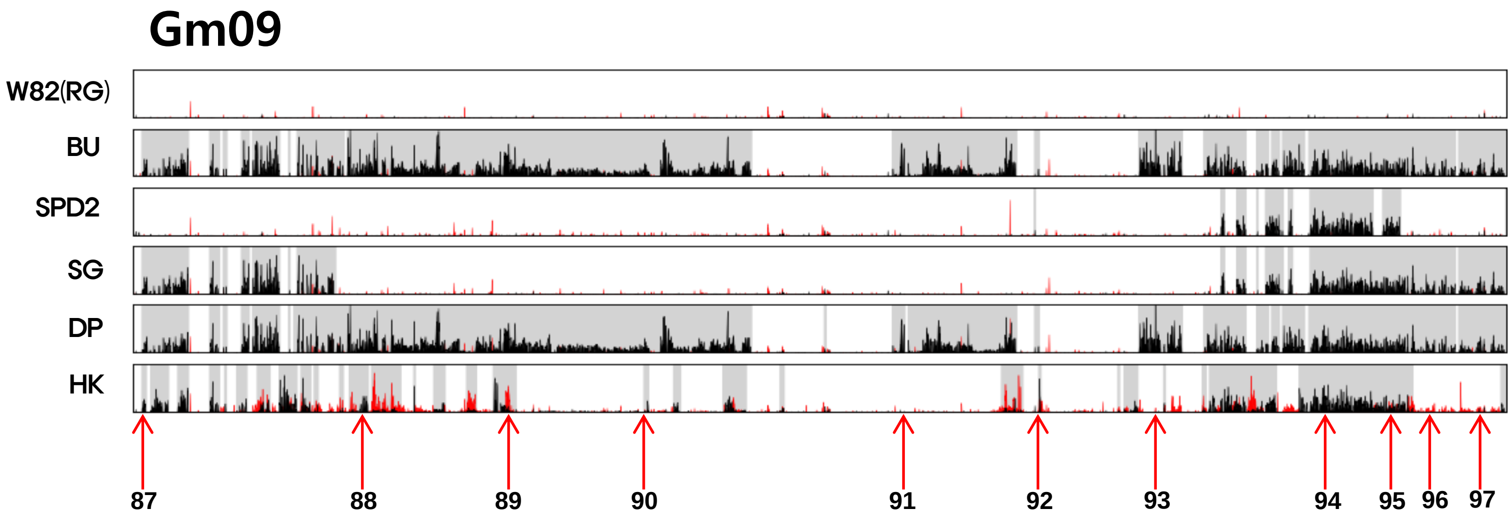

Figure S1. (Continued)

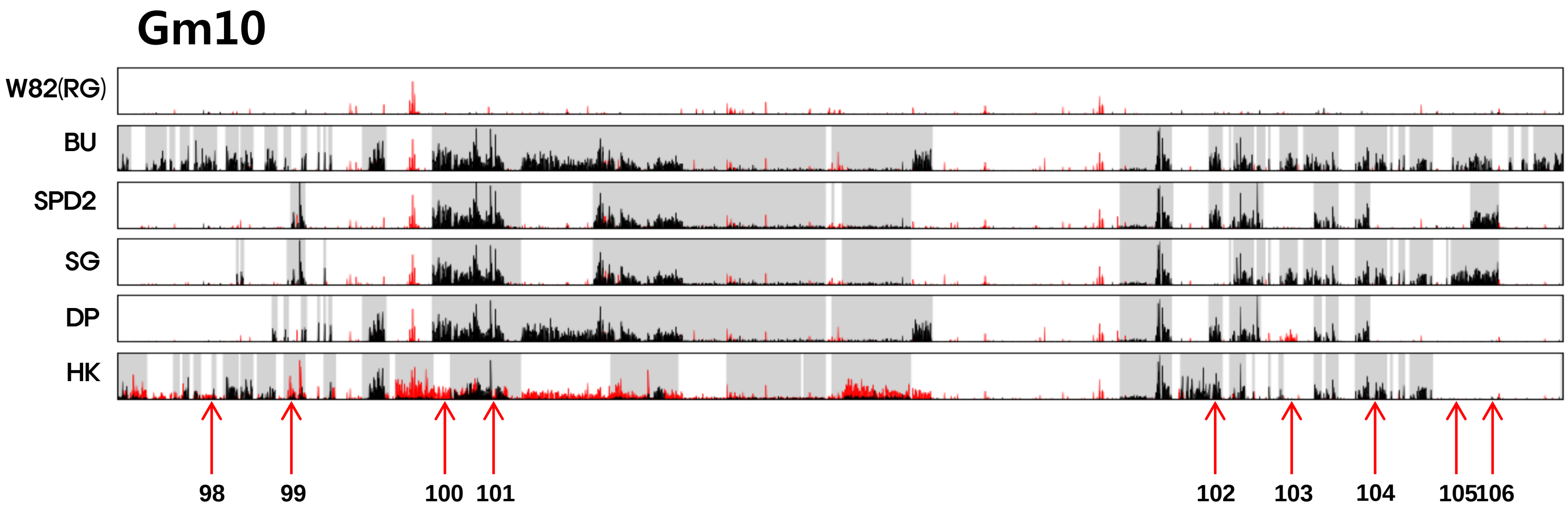

Figure S1. (Continued)

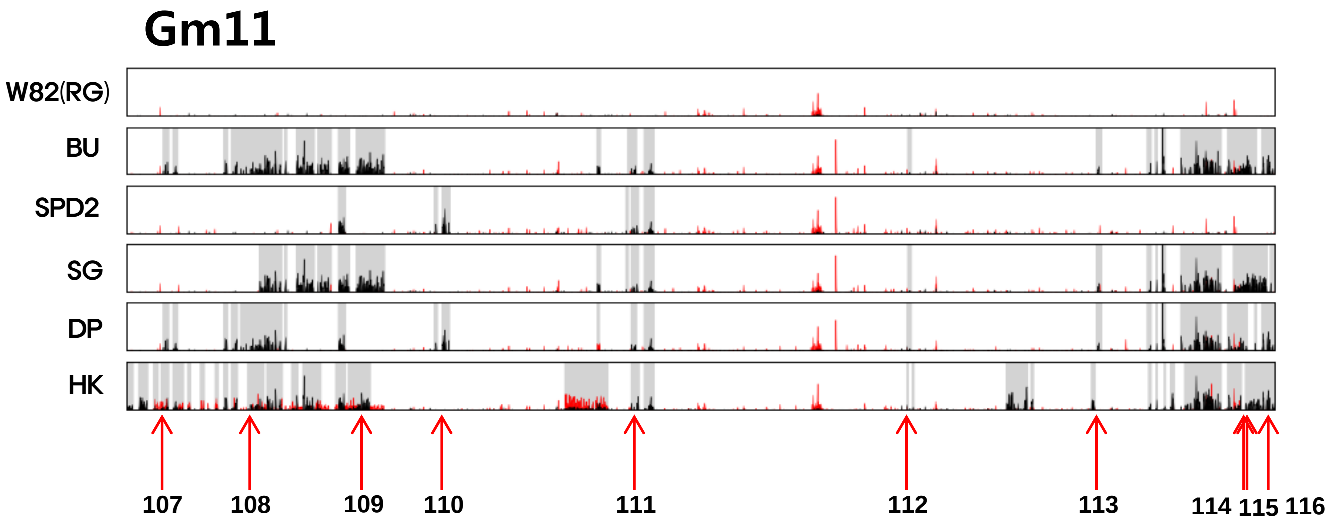

Figure S1. (Continued)

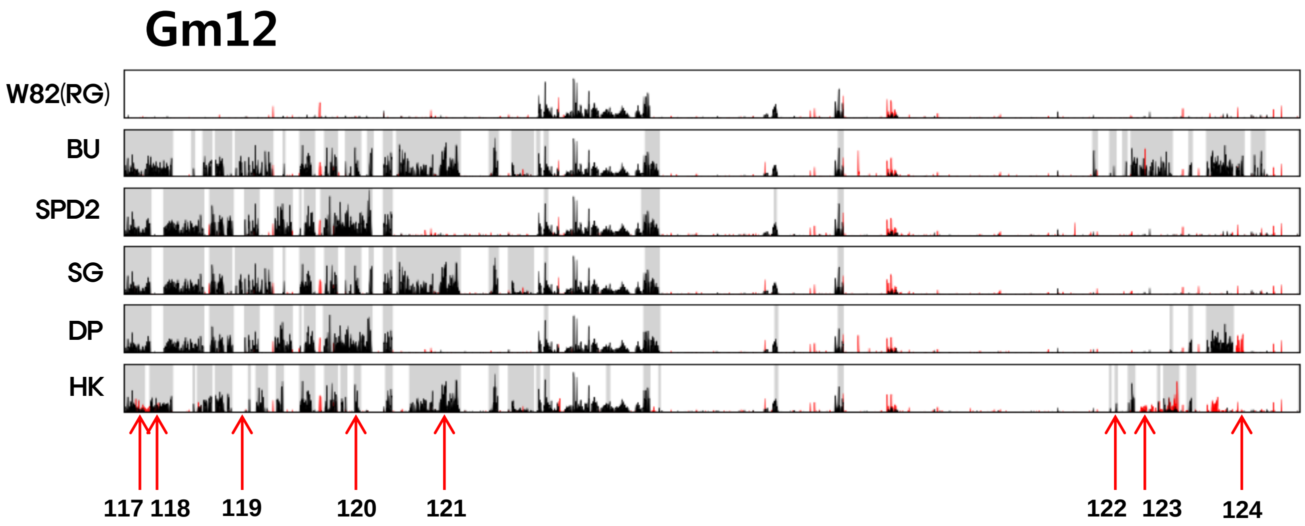

Figure S1. (Continued)

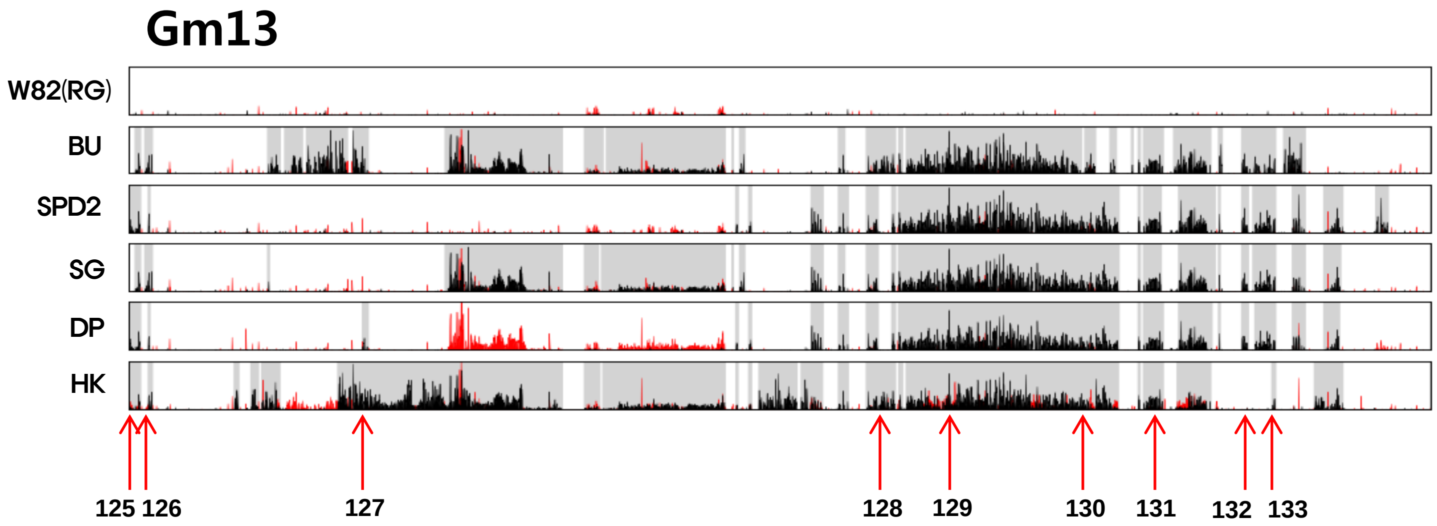

Figure S1. (Continued)

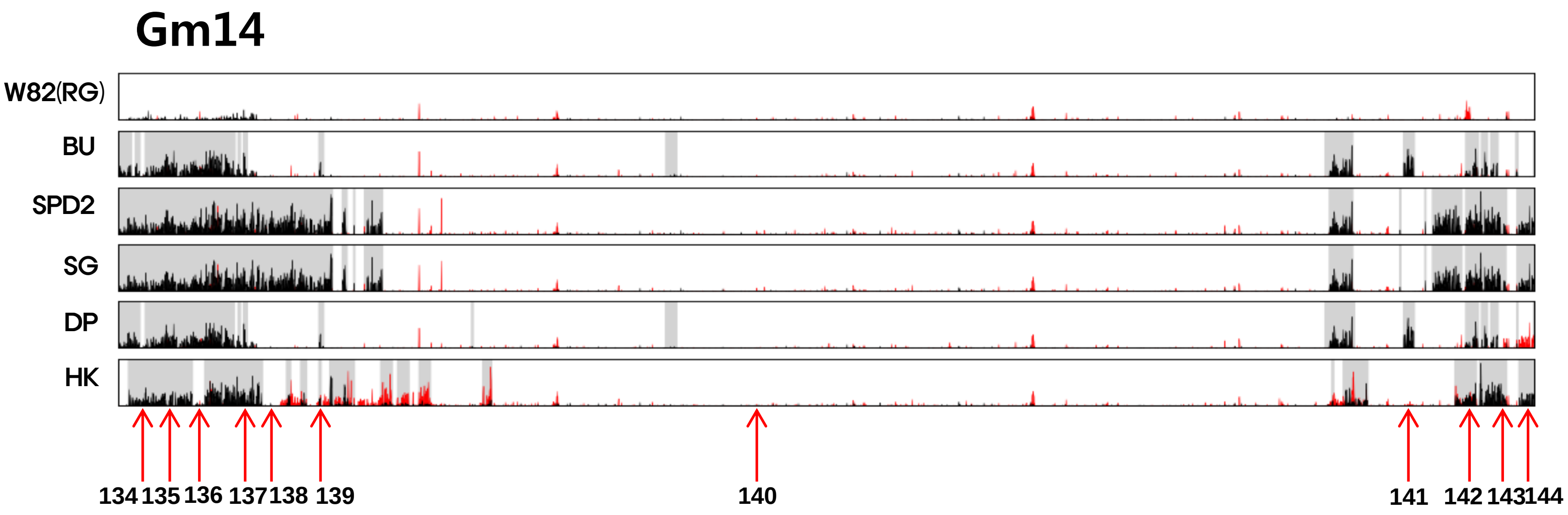

Figure S1. (Continued)

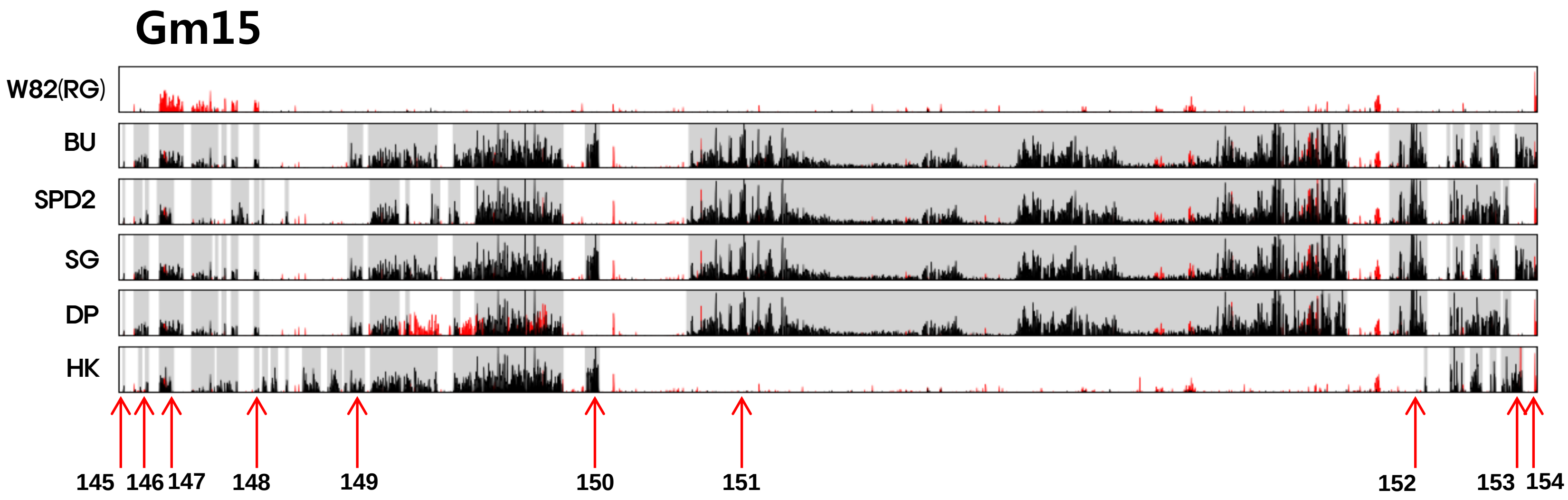

Figure S1. (Continued)

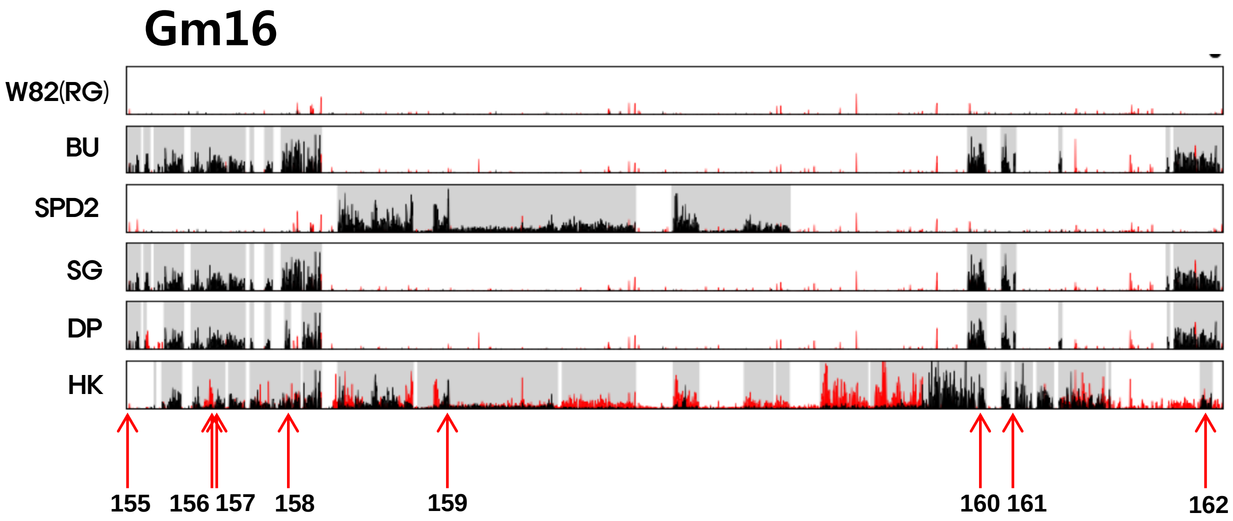

Figure S1. (Continued)

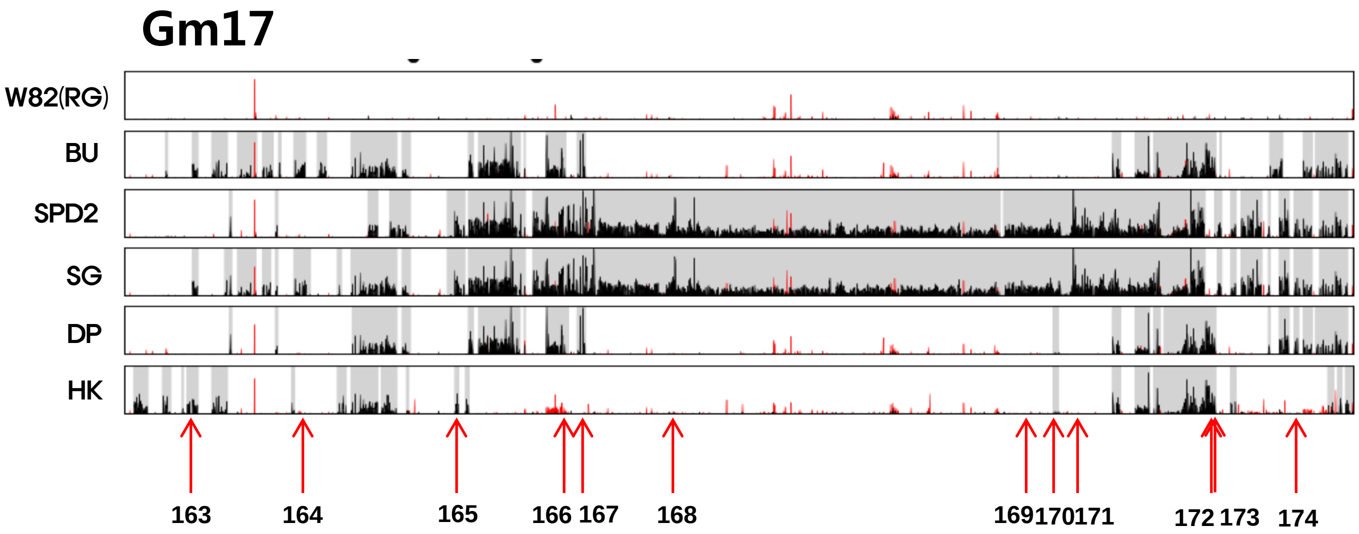

Figure S1. (Continued)

Gm18

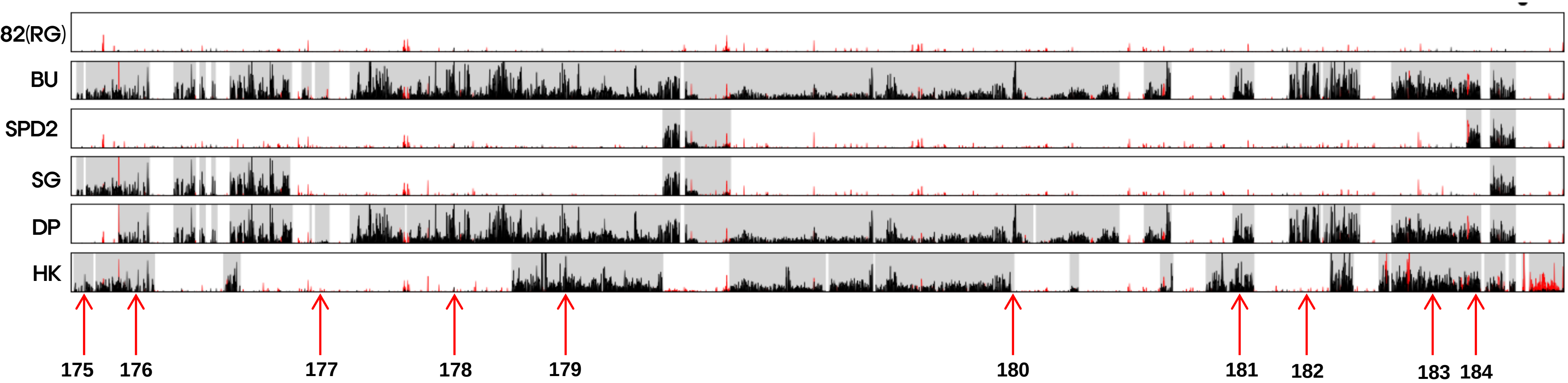

Figure S1. (Continued)

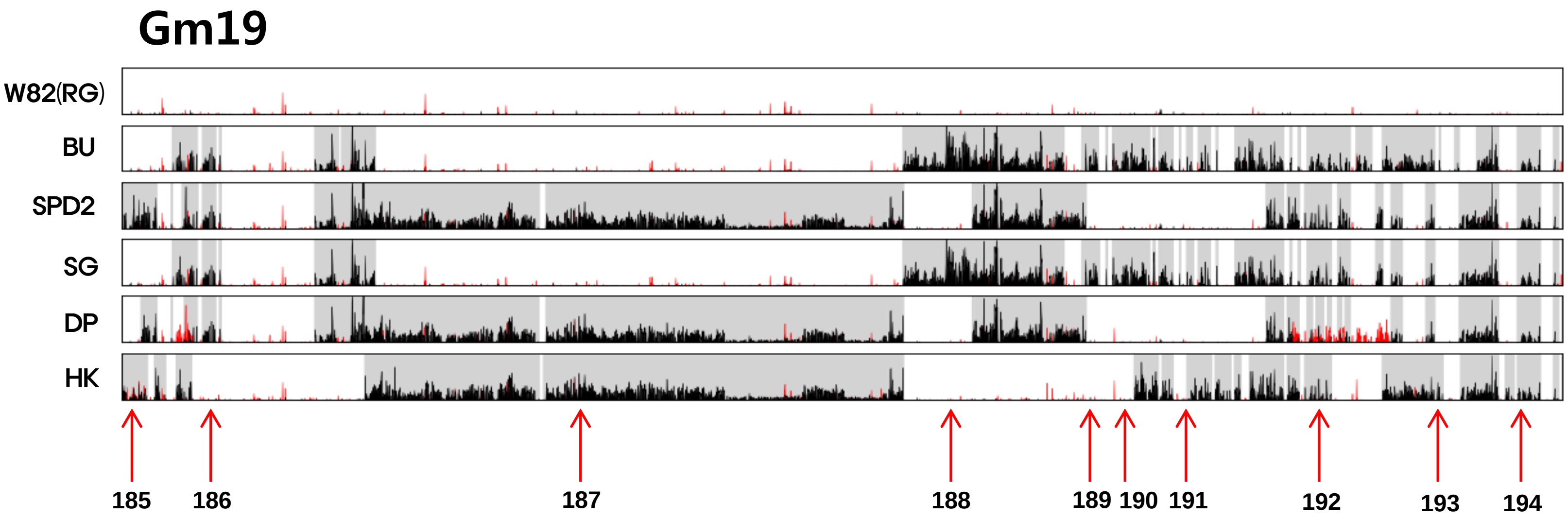

Figure S1. (Continued)

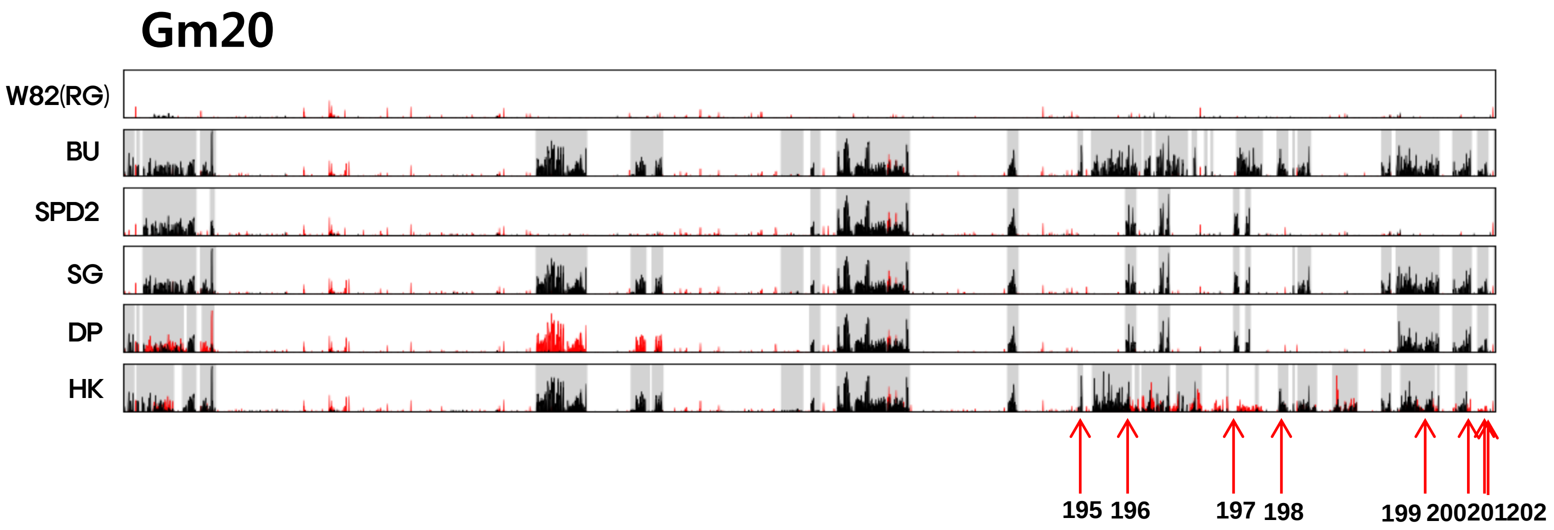

Figure S1. (Continued)
